# FedMCP++: Integrating Modular Expert Heads with Prototype-Guided Contrastive Distillation for Wireless Personalized Federated Learning

**DOI:** 10.3390/s26175328

**Published:** 2026-08-22

**Authors:** Faruk Baturalp Günay, Ferhat Bozkurt

**Affiliations:** Department of Computer Engineering, Faculty of Engineering, Atatürk University, 25240 Erzurum, Turkey; fbozkurt@atauni.edu.tr

**Keywords:** federated learning, personalization, contrastive distillation, modular expert heads, communication efficiency, edge computing

## Abstract

**Highlights:**

**What are the main findings?**

- FedMCP++ equips every client with a complete private model—a lightweight convolutional backbone plus a private expert head—and couples the federation through prototype-guided aggregation and contrastive distillation; clients collaborate solely via soft predictions and class prototypes computed on a small public proxy set, and no model parameters are ever exchanged.
- Across six vision benchmarks and 10/20/30-client settings, FedMCP++ improves average client-level accuracy over non-collaborative local training in the majority of configurations, with the largest gains under severe per-client data scarcity; the effects are dataset-dependent rather than uniform.

**What are the implications of the main findings?**

- Because no parameters are transmitted, the per-round uplink payload is a fixed-size message of soft predictions and prototypes (42.6 KB in our setting), 9.9–12.8× smaller than full-model synchronization and invariant to model capacity.
- Keeping all model weights on the device structurally precludes gradient- and parameter-based reconstruction attacks and supports on-device personalization for Internet of Things and edge deployments; residual output-channel leakage is assessed with the membership-inference protocol.

**Abstract:**

Federated learning (FL) enables collaborative model training across decentralized clients while preserving data privacy, yet real-world deployments still face communication bottlenecks, performance degradation under heterogeneous client data, and limited personalization. In this study, we introduce FedMCP++, a modular and communication-efficient personalized FL framework in which every client owns a complete private model—a lightweight convolutional backbone with a private expert head—and collaboration is carried out entirely through knowledge exchange rather than parameter exchange. In each round, clients share only temperature-softened class predictions and class-wise feature prototypes computed on a small public proxy set; the server fuses them into an accuracy-weighted teacher and broadcasts the result, and clients realign their models through knowledge distillation, an instance-level contrastive objective, and prototype alignment. We evaluate FedMCP++, its ablations, and two knowledge-based baselines on six benchmark vision datasets with 10, 20, and 30 clients. The results indicate dataset-dependent trade-offs rather than uniform superiority: collaborative distillation improves average client-level accuracy over independent local training in twelve of eighteen configurations—most clearly under severe per-client data scarcity (e.g., up to +2.7 percentage points on KMNIST and +2.4 on STL-10 with 20–30 clients)—whereas independent training ensembles remain strongest on SVHN and CIFAR-10 at the studied budgets. Because no parameters are transmitted, the per-round uplink payload is a fixed-size 42.6 KB message, 9.9–12.8× smaller than full-model synchronization, and is invariant to model capacity. These properties make FedMCP++ a flexible framework for personalized FL in wireless edge and Internet of Things environments where bandwidth and privacy constraints are paramount.

## 1. Introduction

In recent years, the widespread deployment of intelligent devices and sensors across diverse applications such as healthcare, finance, and mobile communications has significantly increased the amount of decentralized data generated at the edge of networks [1,2]. Consequently, concerns regarding data privacy, network bandwidth constraints, and stringent regulations have made traditional centralized machine learning paradigms impractical. Federated Learning (FL) has emerged as a promising decentralized learning paradigm designed to collaboratively train global models without exchanging raw data between client devices [3]. Instead, model parameters or updates derived from local training processes are communicated, thereby reducing privacy risks and communication overhead.

Despite the advantages of FL, various challenges persist, particularly those related to statistical data heterogeneity, known as the non-independent and identically distributed (non-IID) data problem [4]. Under non-IID settings, clients possess distinct data distributions, causing divergence among local model parameters and subsequently leading to suboptimal global performance. To overcome this issue, personalized federated learning (PFL) has been introduced, aiming at effectively capturing both global shared knowledge and local-specific information [5]. PFL methods typically involve maintaining client-specific components or layers in addition to a shared global model.

Recently, various modular architectures have been explored within the PFL domain. For example, in FedRep [6], the model was divided into shared and personalized layers, allowing clients to train personalized classifiers independently while benefiting from globally learned representations. Another similar approach, FedPer [7], was introduced, where each client retained local personalization layers atop a globally trained backbone. Such modular structures have demonstrated improved performance and greater flexibility in handling data heterogeneity and enhancing personalization.

The rapid evolution of FL has greatly enhanced the capabilities and breadth of decentralized model training frameworks. A variety of cutting-edge techniques have been proposed to tackle persistent challenges, including data heterogeneity (non-IID distributions), model personalization, and safeguarding data privacy. For instance, FedProx [8] introduced a proximal term into the local objective function to stabilize updates across heterogeneous client environments, significantly improving convergence performance. To further mitigate variance caused by data heterogeneity, SCAFFOLD [9] utilized control variates to efficiently reduce client drift, enhancing stability in FL scenarios. Another notable advancement, FedDF [10], employed ensemble distillation mechanisms on shared public datasets, enabling robust aggregation of local client models without compromising client privacy. In parallel, methods leveraging generative modeling, such as FedGen [11], have emerged, allowing synthetic data generation at clients, thereby improving global model training while strictly preserving data confidentiality. Moreover, techniques that explicitly incorporate regularization, like FedRDS [12], have combined selective data sharing with regularization strategies, achieving better alignment of local and global models and facilitating efficient training. Furthermore, recent research has explored transformer-based models such as FedFormer [13,14], integrating attention mechanisms to adaptively aggregate client information, thereby improving predictive performance under highly heterogeneous scenarios. These innovative methods have significantly broadened FL’s applicability, providing sophisticated solutions that effectively balance accuracy, personalization, communication efficiency, and privacy.

Motivated by these developments, a novel federated learning framework incorporating modular expert heads and contrastive feature distillation has been proposed in this study. In contrast to previous approaches, the proposed framework involves a clear separation of shared and personalized components. Specifically, each client maintains a private expert head, responsible solely for local classification tasks, while contributing to a federation-wide alignment of backbone representations that share a common architecture. Furthermore, knowledge distillation and contrastive learning mechanisms have been introduced to ensure effective alignment between local client representations and the global model, without exchanging sensitive client data or, indeed, any model parameters at all.

It is worth clarifying at the outset how FedMCP++ differs from the most closely related state-of-the-art methods, namely model-contrastive federated learning (MOON) [15] and class-prototype-guided personalization (FedCPG) [16]. MOON derives its contrastive signal solely from a single global-model anchor, which constrains the expressiveness of local representations and provides no explicit class-level alignment. FedCPG employs global class prototypes on top of a lightweight backbone, but it lacks both a contrastive personalization mechanism and modular client-specific components. In contrast, FedMCP++ uniquely combines three elements: (i) a strict decoupling of a collaboratively aligned—but never parameter-averaged—backbone from private, client-specific expert heads; (ii) prototype-guided contrastive distillation performed over a small public proxy set, which aligns local and global feature spaces at both the class level and the instance level; and (iii) parameter-free communication, in which neither the backbone nor the personalized components are ever transmitted: the entire exchange consists of fixed-size soft predictions and prototypes computed on the proxy set.

**Nomenclature:** The name FedMCP++ was coined independently of, and should not be confused with, FedMCP [17], a parameter-efficient federated fine-tuning method for pre-trained language models that aggregates a global adapter and applies a model-contrastive regularizer between a global and a private adapter. Beyond the coincidental acronym, the two methods differ in scope and mechanism: FedMCP [17] fine-tunes frozen language backbones and communicates adapter parameters, whereas FedMCP++ trains compact vision models from scratch and communicates no parameters at all, relying instead on proxy-set predictions and prototypes. The relationship is made explicit here to avoid any ambiguity between the two methods.

The primary contributions of this study are as follows:

- We develop FedMCP++, a flexible and modular framework for federated learning that separates global representation learning from local task adaptation. The architecture integrates a compact convolutional backbone—identical in architecture but private in parameters on each client—with private expert heads, enabling model personalization while keeping every parameter on the device.
- We introduce a contrastive distillation approach that leverages a small public proxy dataset to align client-side and server-side embeddings using an instance-wise NT-Xent loss. Together with an accuracy-weighted teacher and prototype alignment, this promotes representation consistency across clients while keeping raw data and parameters on the device; we give a fully explicit formulation of the loss, including the two projection modules, the positive/negative pair construction, and the temperature.
- We evaluate FedMCP++ across six visual classification benchmarks—MNIST, Fashion-MNIST, KMNIST, SVHN, STL-10, and CIFAR-10—under three federated configurations with 10, 20, and 30 clients. Rather than uniform superiority, the results reveal dataset-dependent trade-offs: parameter-free collaboration lifts client-level accuracy and macro-F1 most clearly under severe per-client data scarcity, whereas independent training ensembles remain strongest on SVHN and CIFAR-10 at the studied budgets. Because the method transmits no parameters at all, its fixed 42.6 KB per-round uplink is 9.9–12.8× smaller than full-model synchronization and invariant to model capacity.
- We conduct a comprehensive ablation study to assess the impact of each component—expert heads, contrastive alignment, and prototype-guided aggregation. The findings are reported as regime-dependent effects: prototype alignment yields its clearest benefit on KMNIST at 20 clients but is counter-productive in the most data-scarce RGB settings, and differences among several variants fall within the empirically measured run-to-run noise envelope, which we quantify and report.

The remainder of this paper is structured as follows. Section 2 reviews related works on federated and personalized federated learning. The detailed architecture and methodology of the proposed modular federated learning framework are introduced in Section 3. The experimental setup and comprehensive results are presented and discussed in Section 4 and Section 5. Finally, Section 6 summarizes the findings and outlines potential future directions.

## 2. Related Work

FL has recently emerged as an effective decentralized machine learning paradigm, allowing multiple clients to collaboratively train a global model without directly exchanging private data. This approach preserves user privacy and reduces communication costs, making it highly appealing for various applications, including healthcare, finance, and edge computing [1,2]. However, FL faces significant challenges, particularly data heterogeneity across clients, which is commonly referred to as the non-IID problem. This heterogeneity can lead to divergence in local model updates, adversely affecting the accuracy and convergence of the global model [4]. Consequently, extensive research has been dedicated to mitigating these effects, employing various personalization and aggregation techniques [3].

PFL has gained attention due to its ability to cater specifically to client-specific data distributions and improve performance under non-IID conditions. For instance, Li et al. [18] introduced FedBN, employing local batch normalization layers to capture individual client characteristics, thus allowing effective model personalization. Similarly, Fallah et al. [19] utilized a meta-learning framework to adapt quickly to the distinct data distributions of each client. While these approaches have improved local adaptability, they often require additional computational overhead and do not explicitly address the alignment of global and local representations.

Modular architectures have subsequently been developed to address these concerns by clearly distinguishing shared and personalized components. FedPer [7], for instance, separates the model into a shared backbone and personalized classification layers, allowing individual clients to benefit from global knowledge while maintaining personalized performance. FedRep [6] similarly disentangles feature extraction from classification, enabling clients to fine-tune their own classifiers on top of shared features. These modular approaches have significantly improved personalization capabilities, but still often neglect the explicit alignment between local and global representations.

To enhance representation learning further, contrastive learning approaches have recently been explored within the federated context. Li et al. [15] introduced MOON, a modelcontrastive learning method that aligns local model representations with the global representation to improve consistency across diverse clients. While MOON has achieved notable performance gains, it relies primarily on the global model for contrastive signals, potentially limiting local model expressiveness and personalization.

In parallel, knowledge distillation (KD) techniques have been adapted for FL to facilitate effective knowledge transfer without exchanging sensitive client data directly. Li and Wang [20] proposed FedMD, which utilizes a public dataset to distill knowledge among heterogeneous client models, thus preserving privacy while improving model generalization. Ensemble-distillation variants such as FedDF [10] fuse client knowledge on unlabeled proxy data at the server, and DS-FL [21] exchanges only proxy-set model outputs to reduce communication to a model-size-independent payload; FedMCP++ belongs to this parameter-free family, which it augments with accuracy-weighted teacher construction, instance-level contrastive alignment, and prototype guidance. Furthermore, recent advancements such as Adaptive Expert Models [22] have leveraged the Mixture of Experts (MoE) framework, dynamically allocating personalized expert models to clients based on their specific data distributions. Despite these innovations, such methods typically incur increased computational complexity, making them less suited for edge-device scenarios.

Additional research has investigated self-distillation techniques, such as FedBSD [23], which encourage models to refine themselves through iterative learning from their own predictions. This approach effectively reduces model drift and enhances local personalization, although it may not adequately leverage global knowledge during the local optimization process. Similarly, edge-level personalization frameworks like FedTP [24], which use transformer-based architectures with personalized self-attention, have been proposed to address resource limitations and data heterogeneity in edge computing environments. However, transformer-based solutions remain computationally demanding for typical edge devices.

To further tackle the challenge of data heterogeneity, clustering-based methods like FedGKD [25] group similar clients and apply intra-group knowledge distillation, thereby enhancing collaborative learning efficiency. Nonetheless, the effectiveness of such methods critically depends on accurate cluster formation, which can be challenging and computationally intensive in practice. Moreover, adaptive aggregation techniques such as FedAFK [26] dynamically weight client updates based on feature relevance, attempting to balance global consistency with local personalization. These methods, while promising, often require careful tuning and significant computational overhead.

Additionally, regularization and limited data-sharing strategies have been proposed to alleviate the adverse effects of non-IID data distributions. FedRDS [12], for example, incorporates explicit regularization terms alongside selective data sharing mechanisms to mitigate client drift and enhance overall model stability. Such methods achieve improved global convergence; however, their reliance on controlled data sharing potentially compromises data privacy to some extent, making them less suitable for highly sensitive applications.

Moreover, various recent federated learning frameworks have attempted to further optimize the trade-off between communication efficiency, privacy preservation, and personalization. For example, FedProx [8] introduces a proximal term to stabilize local updates, effectively controlling divergence due to heterogeneous data. Similarly, SCAFFOLD [9] proposes variance reduction mechanisms through control variates, improving convergence in non-IID scenarios. Nonetheless, these methods primarily focus on convergence and communication efficiency, often neglecting the explicit personalization aspect critical for optimal client-specific performance.

Recent attention has also turned towards hybrid federated learning strategies, combining elements of personalization, privacy-preservation, and efficient aggregation. FedDF [10], for instance, performs ensemble distillation on a shared public dataset, achieving high global model accuracy without compromising local personalization. Yet, FedDF may require substantial computational overhead due to repeated ensemble distillation operations, posing significant challenges in resource-constrained environments typical of federated setups.

Recent advancements in FL have explored diverse strategies to balance personalization, privacy, and communication efficiency. FedGen [11] introduces generative models to synthesize artificial data, enabling enhanced global knowledge transfer without direct data sharing. Despite its ingenuity, this approach’s success hinges heavily on the generative model’s fidelity, posing scalability and quality concerns. Modular personalization methods such as FedRep [6] and FedPer [7] achieve notable performance by decoupling global and client-specific model components. However, they often lack mechanisms for aligning global and local semantic spaces. Contrastive learning-based methods like MOON [15] offer representation alignment benefits but primarily depend on global model anchoring, neglecting client-level privacy and flexibility.

Building on contrastive representation strategies, Qiao et al. [27] proposed MP-FedCL, employing multi-class prototypes to align local embeddings with public and global prototype sets. While effective against data heterogeneity, its design relies on full model sharing and does not support client-specific modules or wireless constraints. Similarly, FedCPG [16] facilitates cross-factory knowledge transfer using global class prototypes and a lightweight backbone for fault detection. Yet, it omits contrastive personalization and expert head modularization critical for nuanced adaptation in dynamic environments.

Emerging paradigms in communication-efficient FL include FedAF [28], which eliminates aggregation by training on collaboratively generated synthetic datasets. Although this improves convergence and robustness, the synthetic data quality remains a limitation. In contrast, PEFT [29] leverages foundation models using prompt and instruction tuning, allowing fine-tuning under resource constraints. However, such methods assume availability of largescale pre-trained models, making them less feasible in lightweight wireless settings. Similarly, FedKD [30] improves generalization via global-to-local distillation but lacks bidirectional personalization and semantic alignment mechanisms. Vertical FL solutions like PraVFed [31] address feature-space heterogeneity by aligning privacy-preserving embeddings, yet do not support client-specific logic or contrastive reasoning. Likewise, Salmeron and Arévalo [32] proposed SquareFL, an interpretable FCM-based FL framework for both horizontal and vertical data partitions. Though effective in structured settings, it relies on symbolic modeling and does not accommodate the lightweight, wireless-focused architecture needed in many real-world FL scenarios.

Personalized FL is also increasingly studied in vehicular edge networks, where statistical heterogeneity interacts tightly with communication constraints. A recent scheme for the Internet of Vehicles (IoV) jointly optimizes communication and model intelligence to tackle long-tailed heterogeneity across vehicles [33], and a comprehensive survey of federated learning for connected and automated vehicles systematizes the data-heterogeneity, privacy, and communication challenges that arise in vehicular deployments [34]. These works reinforce two design requirements that FedMCP++ targets directly: bounded, model-size-independent communication payloads and client-level (rather than only global) performance objectives. In the natural-language domain, FedMCP [17] combines a globally aggregated adapter with a private adapter under a model-contrastive regularizer for pre-trained language models; despite the similar acronym, it addresses a different problem and communicates adapter parameters, whereas the present framework is parameter-free in its exchange (see Section 1).

In contrast to these works, our proposed framework, FedMCP++, introduces a novel architecture that couples per-client lightweight backbones—identical in architecture yet private in parameters—with modular client-specific expert heads. By employing prototype-guided contrastive distillation, it ensures semantic alignment between local and global representations without exposing private client information. This design uniquely supports wireless federated environments by minimizing bandwidth usage, preserving personalization, and enabling interpretable and resource-efficient learning. Unlike synthetic data approaches (e.g., FedAF) or foundation-model-dependent techniques (e.g., PEFT), FedMCP++ maintains a compact architecture that promotes generalization and robustness. Additionally, in contrast to distillation methods that still transmit (compressed) model parameters, such as FedKD [30], our approach removes parameter exchange altogether and shares knowledge exclusively through fixed-size proxy-set outputs.

As highlighted in Table 1, existing federated and personalized learning methods demonstrate various strengths but also exhibit important limitations. Classical algorithms such as FedAvg [35] and FedProx [8] rely on full-model synchronization and lack explicit personalization strategies, making them ineffective in non-IID scenarios. More advanced approaches like FedMA [36], MOON [15], and pFedBayes [37] incorporate alignment or probabilistic modeling, but introduce significant computational complexity. Modular methods such as FedPer [7] and FedRep [6] improve personalization through client-specific heads, yet often neglect global–local semantic alignment. Techniques like pFedLA [38], FedDWA [39], and FedSeq [40] focus on fine-grained or dynamic aggregation but suffer from hyperparameter sensitivity or scheduling complexity. Communication-efficient methods such as FedAF [28] and PEFT [29] reduce bandwidth usage but depend on synthetic data or large-scale pre-trained models, limiting practicality in edge settings. Finally, recent frameworks like HyFDCA [41] and FairDPFL-SCS [42] provide theoretical guarantees and fairness improvements, but are specialized toward convex objectives or require strategic client selection heuristics.

In contrast, our proposed FedMCP++ framework addresses these gaps by integrating a modular architecture with contrastive distillation and prototype-guided alignment. Unlike prior works, FedMCP++ decouples global representation learning from local adaptation via private expert heads, enabling personalization at a per-round communication cost that is fixed by the proxy-set size and class count rather than by the model size. It further enhances alignment through a small public proxy dataset, maintaining privacy while promoting consistent feature spaces across clients. As the experiments in Section 4 show, the resulting behavior is dataset-dependent—client-level metrics benefit most under severe per-client data scarcity—while the communication footprint remains an order of magnitude below parameter-synchronization methods, making the framework attractive for heterogeneous and resource-constrained federated environments.

## 3. Materials and Methods

**Figure 1:**
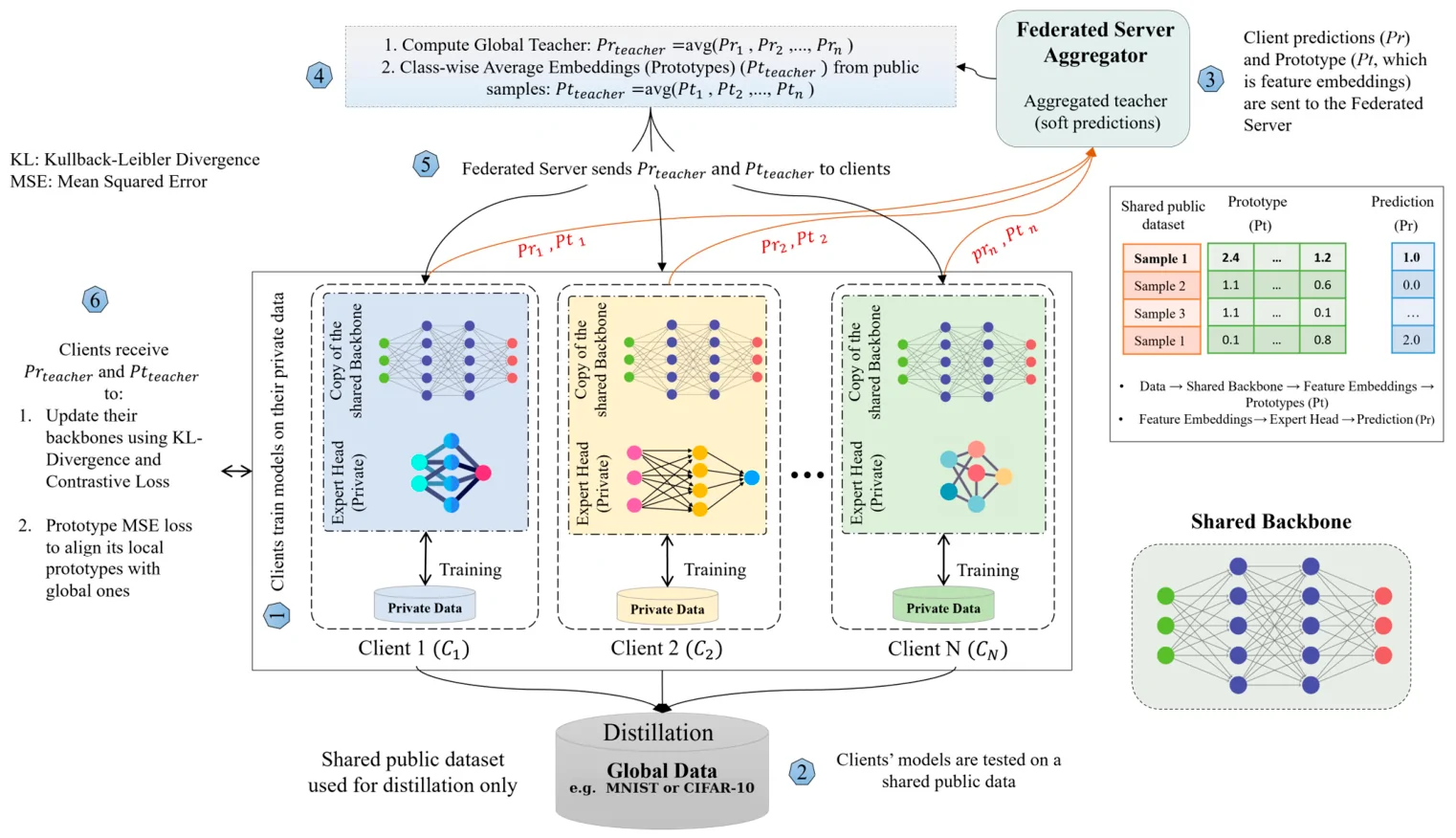
The prototype-guided contrastive distillation workflow of FedMCP++ for wireless personalized federated learning. Clients exchange only soft predictions (Pr) and prototypes (Pt) computed on the public proxy set; the server forms accuracy-weighted teacher aggregates (avg(·) in the figure; Equation (2)) and broadcasts them. No model parameters are transmitted. Colored client panels distinguish the individual clients; red arrows and labels denote the uploaded quantities (Pr, Pt), and the numbered badges mark the workflow steps.

In this section, the proposed federated learning framework is detailed. The architecture is designed to enable personalized, efficient, and privacy-preserving training across heterogeneous clients through the integration of architecturally identical but privately parameterized client backbones, private expert heads, and contrastive knowledge distillation as depicted in Figure 1.

### 3.1. System Overview and Motivation

The core objective of this work is to develop a communication-efficient and personalization-aware federated learning framework, referred to as FedMCP++, suitable for deployment in heterogeneous wireless environments. This framework is explicitly designed to overcome critical limitations in traditional FL settings—such as performance degradation under non-IID distributions and communication bottlenecks—through modularity, knowledge distillation, and prototype alignment. The method operates across a set of *N* clients *C*_1_, C_2_, …, C*_N_* and one central server, with access to a small public dataset *_p_* used only for collaborative alignment. As illustrated in Figure 1, the training process alternates between local supervised learning and global collaborative distillation through soft predictions and feature prototypes.

#### 3.1.1. Workflow Summary

To make the workflow of Figure 1 explicit, each communication round proceeds in six distinct steps. (1) Local supervised training: each client trains its private backbone and expert head on its private data by minimizing the cross-entropy loss in (1). (2) Public evaluation: every client runs its updated model on the shared public proxy set to obtain temperature-softened class predictions and class-wise feature prototypes. (3) Upload to the server: each client transmits only these fixed-size outputs; model parameters, gradients, raw data, expert heads, and projection modules never leave the device. (4) Server aggregation: the server fuses the received predictions into an accuracy-weighted teacher distribution, Equation (2), and averages the prototypes with the same weights into a global prototype matrix. (5) Broadcast: the teacher predictions and global prototypes are sent back to all clients. (6) Local distillation and alignment: each client refines its model in three consecutive stages—logit-level distillation combined with the instance-level contrastive objective of Equation (3) over proxy mini-batches, followed by a single prototype-alignment step—so that, over the course of the round, the four learning signals of the composite objective in Equation (4) are minimized.

#### 3.1.2. Client Architecture and Local Learning

**Figure 2:**
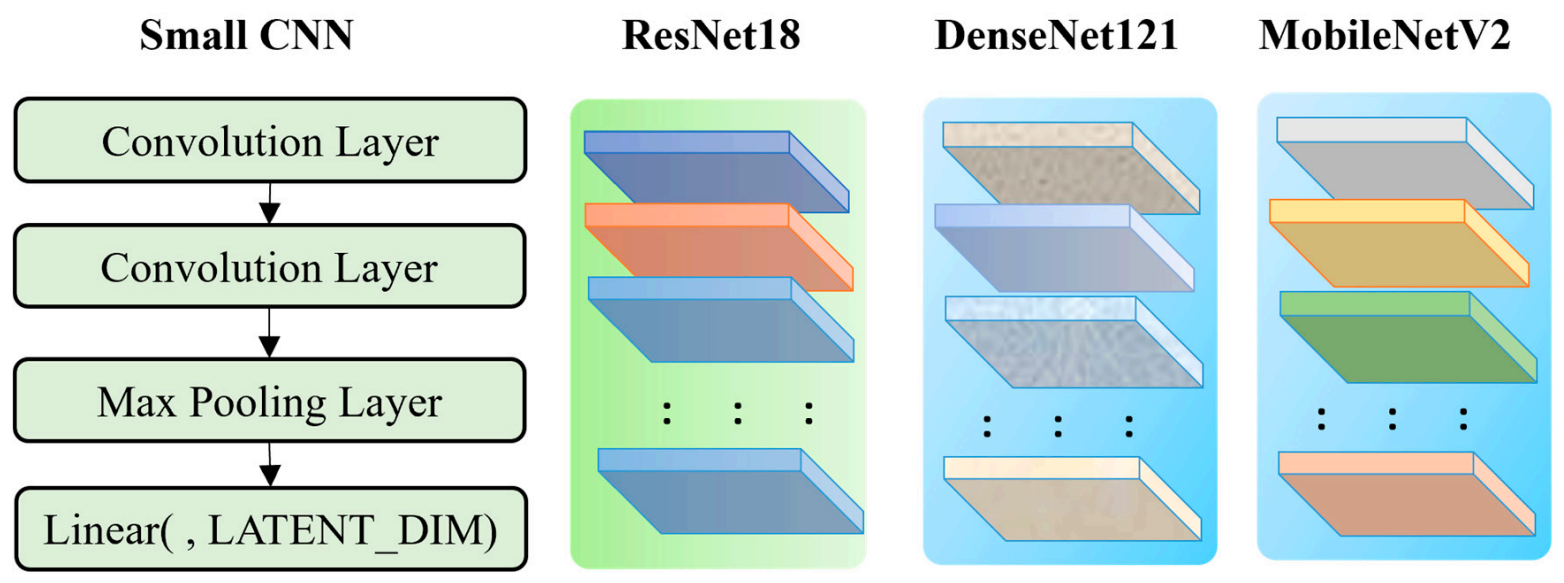
Backbone architecture (identical across clients; parameters remain private to each client).

Each client maintains a lightweight neural model composed of a backbone, a private expert head, and two projection modules. A backbone f*_θ_*, a convolutional neural network (CNN)-based feature extractor. Every client instantiates the same backbone architecture, but the parameters are private: the instances are aligned across clients through the distillation and alignment stages of Section 3.1.5 rather than through parameter averaging. Four light-weight models have been employed as the backbone, and we rely on the small CNN-based model as the default backbone. This choice is technically motivated: its compact size (only 105.22 K–136.22 K parameters depending on the input resolution, as reported in Table 6) keeps on-device computation, memory, and energy within edge-device budgets—the per-round communication payload itself is independent of the model size in our protocol (Section 5.1)—and it trains roughly six times faster than deeper alternatives such as DenseNet121 while remaining within about half a percentage point of their accuracy on MNIST (Table 3). Both properties are critical requirements for battery-powered devices in wireless edge networks. The architecture is depicted in Figure 2; it maps each input to a 64-dimensional embedding that is ℓ2-normalized before further processing. *A* private expert head *h_i_*, a classifier unique to each client that enables personalization. Two lightweight linear projection modules P*_s_* and *P_t_*: the former, ℝ^64^→ℝ^32^, is applied to backbone embeddings and the latter, ℝ^10^→ℝ^32^, to teacher prediction vectors, mapping both into a common 32-dimensional space for the instance-level contrastive alignment of Section 3.1.5. During each round, a client *C_i_* performs local training over its private dataset D*_i_* by minimizing the following supervised loss, Equation (1), where CE(·) denotes cross-entropy loss:

(1) $$L_{sup}^{\left(i\right)}=E_{\left(x,y\right)∼D_{i}}\left[CE\left(h_{i}\left(f_{\theta }\left(x\right)\right),\;y\right)\right]$$

where CE(·) denotes the cross-entropy loss, *h_i_* is the private expert head of client *i*, *f_θ_* is client i’s private backbone—one instance per client, with the client index omitted from fθ for notational brevity—and D*_i_* is the private dataset of client *i*.

#### 3.1.3. Public Evaluation: Soft-Prediction and Prototype Collection

Upon completion of local training, each client evaluates its model on the shared public dataset D*_p_* to produce two outputs. Soft predictions p*_i_*: the temperature-scaled softmax of the expert-head outputs, *p_i_* = softmax(*h_i_*(*f_θ_*(*x*))/*T*), representing soft class probabilities (not raw logits) over public samples, softened by a temperature T > 1. Prototypes Pt*_i_*: class-wise average feature embeddings *μ_c_* = E*_x_*_∈D*c*_ [*f_θ_*(*x*)] computed using the backbone only, where D*_c_* is the subset of public samples of class *c*.

#### 3.1.4. Federated Server Aggregation

The server receives the soft predictions and prototypes of every client and fuses them with accuracy-dependent weights. Let a_i_ denote the accuracy of client i’s predictions on the labelled proxy set. The teacher distribution is the weighted average of Equation (2), and the global prototype matrix Pt_T_ is formed with the same weights, Pt_T_ = Σ_i_ ω_i_ Pt_i_. Weighting by proxy accuracy damps the influence of clients whose models are momentarily weak; this is the principal difference from the uniform averaging used by the EnsKD baseline of Section 4.3. The resulting teacher quantities act as “soft supervisors” broadcast back to all clients:

(2) $$p_{T}\left(x\right)=\sum_{i=1}^{N}\omega_{i}p_{i}\left(x\right),\;\omega_{i}=\frac{a_{i}}{\sum_{j=1}^{N}a_{j}}$$

#### 3.1.5. Distillation and Feature Alignment

Each client then performs a three-stage alignment procedure. *Stage 1—Soft-prediction distillation:* the client matches its softened prediction distribution to the teacher using the temperature-scaled Kullback–Leibler (KL) divergence T^2^·KL(p_i_ ‖ p_T_). *Stage 2—Contrastive representation matching:* an instance-level contrastive loss aligns backbone embeddings with the teacher’s belief vectors. For a proxy mini-batch B, let z_j_ denote the ℓ2-normalized backbone embedding of sample x_j_ and p_T_(x_j_) the teacher distribution for the same sample. The two projection modules map these heterogeneous quantities into a common space, u_j_ = P_s_(z_j_)/‖P_s_(z_j_)‖_2_ and v_j_ = P_t_(p_T_(x_j_))/‖P_t_(p_T_(x_j_))‖_2_, and the loss is the InfoNCE objective of Equation (3),

(3) $${L_{CL}}^{\left(i\right)}=-\frac{1}{\left|B\right|}\sum_{j\in B}log\frac{exp\left({u_{j}}^{⊤}v_{j}/\tau \right)}{\sum_{k\in B}exp\left({u_{j}}^{⊤}v_{k}/\tau \right)}$$

where τ is the contrastive temperature (τ = T = 1.5 in all experiments), the positive pair of sample j is its own teacher projection v_j_, and the remaining |B| − 1 teacher projections in the mini-batch act as negatives. Stages 1 and 2 are optimized jointly, mini-batch by mini-batch, over the proxy set, with weight λ on the contrastive term. *Stage 3—Prototype alignment:* after the distillation pass, the client recomputes its class prototypes on the proxy set and takes a single gradient step on the mean squared error to the global prototypes, α‖Pt_i_ − Pt_T_‖_2_^2^. The composite objective that these stages minimize over a round is the unified four-signal objective of Equation (4)—local supervision, logit-level distillation, instance-level contrastive alignment, and class-level prototype alignment:

(4) $$L_{total}^{\left(i\right)}=L_{CE}^{\left(i\right)}+T^{2}\;KL\left(P_{i}\|P_{teacher}\right)+\lambda \;L_{CL}^{\left(i\right)}+\alpha {\left\|{Pt}_{i}-{Pt}_{teacher}\right\|}_{2}^{2}$$

Here, the first term is the local supervised cross-entropy loss of client i, Equation (1), which is minimized during the local-training phase. The second term is the logit-level distillation loss of Stage 1: the KL divergence between the client’s softened predictions and the accuracy-weighted teacher of Equation (2), scaled by T^2^ to preserve gradient magnitudes under temperature smoothing. The third term is the instance-level InfoNCE contrastive loss of Equation (3), weighted by λ, and the final term is the prototype-alignment loss of Stage 3—the mean squared error between local and teacher prototypes—weighted by α. Equation (4) is thus a four-signal composite objective. Its components correspond to items (a) and (e)–(g) of Algorithm 1 and are optimized in the staged schedule described above—supervised training first, then joint distillation with contrastive alignment over proxy mini-batches, then a single prototype-alignment step—rather than in one joint gradient update; this staged schedule is exactly what the released implementation executes.
**Algorithm 1.** FedMCP++: modular FL with private experts, prototypes and contrastive distillation
**Require:** clients *C*_1_, …, *C**_N_*****; public dataset D*_p_*; local datasets D*_i_*; rounds *R*; epochs *E*; temperature *T*; trade-offs *λ*, *α***
**Ensure: backbones f*_θ_* (one per client; architecture shared, parameters private); private expert heads h***_i_*1:Initialize *f**_θ_***
**and {*h****_i_*}*_i_*_=1_*^N^*2:**for** *r* = 1 **to** *R* **do**3: **for** each client *i* **in** parallel **do**4:  **(a) Supervised update:** train *f**_θ_*****, *h****_i_* on D*_i_* for *E* epochs via cross-entropy, Equation (1)5:  **(b) Public embeddings and soft predictions:** compute *P**_i_*****(*x*) = softmax(*h****_i_*(*f**_θ_*****(*x*))/*T*) and *z****_i_*(*x*) = *f**_θ_*****(*x*) for all *x* ∈ D*_p_***6:  **(c) Local prototypes:** for each class *c*, *Pt**_i_*****[*c*] = (1/|{*x*: *y* = *c*}|) Σ*_y_*_=*c*_ *z****_i_*(*x*)7: **end for**8: **(d) Server aggregation:** *Pteacher = Σi ωi Pi***; *Ptteacher = Σi ωi Pti, with ωi = ai/Σj aj (proxy-accuracy weights; Equation (2))***9: **for** each client *i* **in** parallel **do**10:  **(e) Logit distillation:** L_KD_^(*i*)^ = *T*^2^ KL(*P**_i_*** **‖*P*_teacher_)**11:  **(f) Contrastive alignment:** L_CL_^(*i*)^ = InfoNCE(Ps(zi(x)), Pt(Pteacher(x))); Equation (3)12:  **(g) Prototype alignment:** L_proto_^(*i*)^ = *α* ‖*Pt**_i_***
**− *Pt*_teacher_‖_2_^2^**13:  update fθ and hi via (e)+(f) on proxy mini-batches, then one gradient step of (g); Equation (4)14: **end for**15:**end for**16:**(h) Global inference (evaluation protocol; Section 3.1.6): ŷ = arg max (1/N) Σi softmax(hi(fθ(x))/T)**

#### 3.1.6. Global Evaluation and Ensemble Inference

Global performance is measured with a soft-prediction ensemble evaluation protocol: for every test sample, the temperature-softened predictions of all N client models are averaged and the arg-max is taken, so that the global metric reflects the joint knowledge of the federation without any exchange of model parameters. We emphasize that this is an evaluation construct executed by the simulation server, which holds all client models in memory. In a physical deployment, the same protocol corresponds to each client returning one C-dimensional soft-prediction vector per query (40 bytes per sample for C = 10 classes in float32); alternatively, any single client model can be used locally at zero communication cost, at the client-level accuracy reported as CAcc. The communication accounting in Section 5.1 covers both regimes. Soft probability averaging (rather than hard voting) is used because it matches the temperature-softened quantities exchanged during distillation and remains well behaved for small federations, where hard-vote ties are frequent.

#### 3.1.7. Privacy and Efficiency Considerations

At no point are raw data, labels, gradients, or any model parameters transmitted; the entire training-time payload consists of soft predictions and class prototypes computed on the public proxy set. Two consequences follow. First, attacks that reconstruct private inputs from shared gradients or weight updates, such as gradient-inversion attacks [43], are inapplicable by construction, because the quantities they require never leave the device. Second, the residual disclosure channel is limited to model outputs on public inputs; the extent to which such outputs leak membership information about private training data is an empirical question, which we assess with a confidence-based membership-inference evaluation [44,45] in the revision experiments (Section 5.2). We therefore characterize the privacy profile as the structural elimination of parameter- and gradient-based leakage combined with a bounded, auditable output channel, rather than as an unqualified guarantee. The framework additionally offers a low, model-size-independent communication cost and personalization through the private expert heads.

### 3.2. Training Procedure

The full training process is detailed in Algorithm 1. Clients alternate between local supervised learning, collaborative distillation guided by the weighted teacher, and feature-prototype alignment; the procedure repeats over R rounds. Each round begins with local supervised training (steps 3–7, item (a)), where every client optimizes its private backbone and expert head by minimizing the cross-entropy loss of Equation (1). After fitting to private data, clients evaluate on the small public set, extracting softened predictions and backbone embeddings (item (b)), from which class-wise prototypes are computed by averaging the embeddings of all public samples of each class (item (c)).

On the server (item (d)), the per-client public predictions and prototypes are fused into the accuracy-weighted teacher of Equation (2). Clients then enter the distillation-and-alignment phase (steps 9–14): on each proxy mini-batch they jointly minimize the logit-level distillation term (item (e)) and the InfoNCE contrastive term of Equation (3) (item (f)), after which a single gradient step aligns their prototypes with the teacher prototypes (item (g)). Jointly, these staged updates realize the four-signal composite objective of Equation (4), aligning local feature spaces and output distributions to the federation consensus while preserving personalization via the private heads. Finally, for global evaluation, a virtual predictor is formed by averaging all client classifiers’ softened outputs (item (h); Section 3.1.6).

This design ensures that no model parameters are communicated at all: knowledge flows exclusively through fixed-size proxy-set outputs, whereas backbones, expert heads, and private feature distributions remain on-device. The use of a public dataset for distillation confines the disclosure channel to model outputs on public inputs (Section 3.1.7), and the combined logit-, contrastive-, and prototype-level alignment fosters both cross-client consistency and personalization under per-client data scarcity.

### 3.3. Hyperparameter Setup

Table 2 presents the results of various optimization algorithms applied to a fixed lightweight CNN model. The Stochastic Gradient Descent (SGD) optimizer achieves the best global and client-level scores, confirming its suitability for stable training in federated environments. While adaptive optimizers like Adam and AdamW provide competitive performance, they slightly underperform compared to SGD in this setup. RMSprop and Adagrad show relatively lower scores, indicating that their adaptive mechanisms may not offer significant benefits in this constrained model setting.

Table 3 analyzes how changing the backbone architecture influences model performance when using the SGD optimizer. While advanced pretrained models such as DenseNet121 and MobileNetV2 outperform the custom small CNN in raw metrics, the small CNN remains a competitive option due to its low complexity and rapid training time. This efficiency makes it ideal for deployment in real-time and low-resource federated learning settings such as edge computing or IoT (Internet of Things) systems. After a comprehensive investigation of the hyperparameter options, the best-performing configurations were identified based on the results presented in Table 2 and Table 3. Accordingly, the final hyperparameters adopted across all experiments are summarized in Table 4. The federated setting consists of 10, 20, and 30 clients, each performing 1 local epoch per communication round for a total of 15 rounds. A mini-batch size of 64 was used, and stochastic gradient descent (momentum 0.9) served as the optimizer for both the backbone and the expert head; all remaining fixed settings—including the prototype weight α, the contrastive temperature τ, the proxy-set size, the participation scheme, the partitioning scheme, and the random seed—are listed in Table 4 for full reproducibility.

### 3.4. Evaluation Metrics

To thoroughly assess the performance of the proposed federated learning framework, a comprehensive set of evaluation metrics is employed. These metrics offer both global and client-level insights into classification performance, robustness, and generalization, especially under non-IID conditions. The evaluation metrics are Accuracy, Precision, Recall (Sensitivity), and F1-Score. All metrics are computed using macro-averaging across all classes. This approach treats each class equally, regardless of its frequency, and is preferred in imbalanced or multi-class federated learning tasks. In all experiments, both client-level and global metrics are reported. For global evaluation, predictions are aggregated across all clients using softmax averaging over all client models (the ensemble protocol of Section 3.1.6). For client evaluation, each client’s performance is computed individually and then averaged across the federation. In addition to these averages, the revision protocol also records client-level dispersion statistics—the standard deviation of client accuracies, the worst-client accuracy, and the best-to-worst gap—as fairness indicators (Section 5.2); they are computed per configuration from the individual client metrics. For label-skewed partitionings, client-level accuracy is additionally reported under two complementary protocols: on the full test split, which scores each client model as a general-purpose classifier, and reweighted by the client’s private label distribution, which scores each model on its own deployment distribution; the two protocols answer different questions and can rank personalized and consensus-based methods differently.

## 4. Results

### 4.1. Dataset Overview and Usage

To comprehensively evaluate the proposed federated learning framework, we utilized six publicly available vision datasets with varying complexity, domains, and resolutions. Table 5 provides an overview of each dataset’s characteristics and its role in FL research. We evaluate FedMCP++ across six publicly available vision datasets that are MNIST, Fashion-MNIST, KMNIST, SVHN, CIFAR-10, and STL-10 as shown in Table 5.

Each training set was partitioned among the clients by uniform random sampling without replacement into equal-sized disjoint shards (⌊n/N⌋ samples per client). This partitioning is label-homogeneous in expectation; the experimental difficulty therefore stems from per-client sample scarcity—which grows sharply with the federation size—together with the finite-sample label skew it induces, rather than from an explicitly engineered label-distribution shift. We state this explicitly because the original submission loosely described the shards as non-IID; genuinely label-skewed Dirichlet partitions [46] are supported by the released implementation and are examined in the revision experiments (Section 5.2). Concretely, each client holds 6000/3000/2000 training samples on the 60,000-image benchmarks (MNIST, Fashion-MNIST, KMNIST), 7325/3662/2441 on SVHN, 5000/2500/1666 on CIFAR-10, and only 500/250/166 on STL-10 for the 10-, 20-, and 30-client settings, respectively.

For each benchmark, the public proxy set consists of 1000 samples drawn uniformly at random from the held-out test split of the same dataset; it is therefore disjoint from every client’s private training shard and shares the native input domain and label space of the task. The proxy set is used exclusively for computing soft predictions, feature embeddings, and class prototypes during the collaborative distillation and alignment phase. Because the final metrics are computed on the full test split, 10% of the evaluation samples are also seen—unlabelled, and only through model outputs—during distillation; the released implementation provides a switch that excludes the proxy samples from the evaluation split so that this overlap can be removed entirely. This benchmark selection allows us to examine performance and robustness across multiple federated configurations.

### 4.2. Model Complexity and Deployment Overhead

Table 6 reports the floating-point operations (FLOPs*_k_*) and parameter counts (Params*_k_*), each in thousands, for all six vision benchmarks. Three “small” datasets (MNIST, Fashion-MNIST, KMNIST) share an identical backbone complexity of 1.12 M FLOPs and 105.2 K parameters, while the three “larger” datasets (SVHN, STL-10, CIFAR-10) uniformly require 1.75 M FLOPs and 136.2 K parameters. Crucially, these figures hold across all compared methods—the Local and EnsKD baselines defined in Section 4.3 and all FedMCP++ variants—since the same backbone architecture is used throughout. The extra modules introduced by FedMCP++ (expert heads, projection layers, prototype buffers) contribute less than 2% to total parameter count and a negligible fraction of FLOPs, ensuring that no method imposes additional backbone overhead.

This uniform complexity profile has two key implications. First, it guarantees *fairness* in runtime and memory comparisons: any observed performance differences (accuracy, F1, fairness) can be attributed solely to algorithmic innovation, not to larger models. Second, it underscores the *practical deployability* of FedMCP++ on edge devices; by matching the compute budget of a plain locally trained model, the additional mechanisms (contrastive distillation and prototype sharing) integrate into existing federated pipelines without hardware upgrades. In summary, Table 6 confirms that all compared configurations share an identical backbone budget, so the differences reported below are attributable to the collaboration mechanisms rather than to model capacity.

### 4.3. Results with Knowledge-Based Baselines and Ablations

Table 7, Table 8 and Table 9 report the evaluation of FedMCP++ and its ablated variants against two knowledge-based baselines on six vision benchmarks with 10, 20, and 30 clients, using global (ensemble) and client-level accuracy and macro-averaged F1. Two baseline labels are corrected relative to the original submission, following a full audit of the released implementation. Local denotes independent local training: clients never communicate during training, and only the ensemble evaluation protocol of Section 3.1.6 combines their predictions (this baseline was previously labelled “FedAvg”, although it performs no parameter averaging). EnsKD denotes ensemble distillation in the spirit of FedMD [20] and FedDF [10]: clients distill from the unweighted mean of all proxy-set predictions (previously labelled “FedKD”). Head-to-head comparisons with parameter-averaging methods—true FedAvg [35], FedPer [7], and FedRep [6]—are reported in Section 4.4. The variant previously labelled “w/o heads” is architecturally identical to the full configuration, because the expert head is private in every variant of this parameter-free design; its rows are therefore relabelled replicate and serve as duplicate executions that quantify run-to-run variation (Section 5.2). Finally, the MNIST row of the full model in Table 7 has been corrected to the values produced by the released notebooks; the originally reported row could not be reproduced from the saved outputs. An independent rerun of this cell with the released implementation on separate hardware yields a global accuracy of 0.9883—within the grayscale noise envelope of both the corrected and the originally printed values—so the rerun cannot arbitrate between them; the correction rests on the archived outputs, with which the rerun is consistent.

With 10 clients, the six configurations are separated by narrow margins on the grayscale benchmarks: on MNIST, all methods lie within 0.5 percentage points (pp) of one another on every metric, and the largest client-level advantage of collaboration appears on KMNIST, where the full model and its variants exceed the Local baseline by roughly 1.4 pp in CAcc (e.g., 0.8897 vs. 0.8756). On the RGB benchmarks, the ranking reverses for the global metrics: Local attains the highest GAcc on SVHN (0.7085) and CIFAR-10 (0.5993), and EnsKD the highest CAcc on SVHN (0.6558). Taken together with the run-to-run noise envelope established by the replicate rows—differences of up to 0.73 pp GAcc on SVHN between two executions of the identical configuration (Section 5.2)—these observations indicate that at the 10-client scale most pairwise differences on the RGB tasks are not individually meaningful, and that the value of the alignment machinery emerges at larger federation sizes.

The ablation rows should accordingly be read as dataset-dependent effects rather than uniform contributions. Removing the contrastive term or the prototypes changes the 10-client results by amounts that mostly lie within the replicate noise envelope, and in several cells an ablated variant is nominally the best (e.g., “w/o protos” attains the highest GAcc on MNIST and the highest CAcc on Fashion-MNIST). A uniformly positive contribution of every component at this scale is therefore not claimed; the component-level reading is deferred to the joint analysis of Table 7, Table 8 and Table 9 in Section 5.

Table 8 presents the 20-client results, where per-client data budgets halve relative to Table 7. On MNIST, the six configurations remain within 0.1 pp of one another. The clearest collaborative gains appear on the grayscale tasks: relative to Local, the full model improves CAcc by 2.20 pp on KMNIST (0.8619 vs. 0.8399) and by 0.63 pp on Fashion-MNIST, and on STL-10 by 2.39 pp (0.2859 vs. 0.2620) with a 3.31 pp margin in CF1. SVHN is also the setting in which the accuracy-weighted teacher shows its most visible advantage over uniform averaging: the full model exceeds EnsKD by 1.34 pp GAcc (0.5787 vs. 0.5653) and 0.93 pp CAcc, while Local retains the best global metrics (0.5946 GAcc). On CIFAR-10, Local leads on all four metrics at this scale.

In summary, the 20-client results show a consistent division of labour: parameter-free collaboration reliably lifts client-level metrics where per-client data are scarce (KMNIST, Fashion-MNIST, STL-10), whereas independent training ensembles remain strongest on SVHN and CIFAR-10 global accuracy. Differences among the FedMCP++ variants themselves remain small; the largest single component effect at this scale is the drop of the “w/o protos” variant on KMNIST (−1.37 pp GAcc and −1.86 pp CAcc relative to the full model), which is the clearest instance in our study of prototype alignment providing a benefit beyond the noise envelope.

Table 9 summarizes the 30-client setting, the most data-scarce regime in our study. On MNIST, all six configurations lie within 0.1 pp. The strongest collaborative margins over Local again occur on the grayscale tasks (KMNIST: +2.74 pp CAcc, 0.8426 vs. 0.8152; Fashion-MNIST: +0.52 pp) and on the STL-10 client-level metrics (+1.67 pp CAcc). Conversely, on SVHN and CIFAR-10 the Local baseline attains the best values on all four metrics, with margins (e.g., +2.84 pp GAcc on SVHN over the full model) that exceed the noise envelope; at 30 clients on these two benchmarks, the distillation signal derived from weak intermediate models appears to regularize clients toward a consensus that is inferior to their independent ensemble.

The 30-client ablations also contain the clearest counter-example to a uniformly positive reading of the components: on STL-10 and CIFAR-10, removing prototype alignment improves upon the full model (+3.24 pp GAcc on STL-10, 0.3530 vs. 0.3206, and +1.53 pp CAcc on CIFAR-10). A plausible mechanism is that, with only 166–1666 samples per client, the class prototypes computed on the proxy set by weak individual models are noisy, and pulling every client toward their weighted average over-regularizes representations on visually diverse RGB data. Prototype alignment is therefore best regarded as a regime-dependent component—beneficial on grayscale tasks at moderate scale (Table 8) and counter-productive in the most data-scarce RGB settings—which the modular design allows practitioners to disable without touching the rest of the pipeline.

### 4.4. Comparison with Parameter-Averaging Personalized FL Baselines

The ablations above cannot substitute for a direct comparison with representation-decoupling PFL methods, because FedMCP++ exchanges no parameters, whereas FedPer [7] and FedRep [6] synchronize a shared backbone by averaging. The released implementation therefore adds faithful re-implementations of true FedAvg [35] (averaging the full model), FedPer (averaging the backbone while heads remain private), and FedRep (as FedPer, with the head updated before the backbone in each local epoch), trained under the identical backbone, optimizer, 15-round budget, and partitioning as all other methods, and initialized from a common broadcast model in accordance with their original protocols (expert heads remain locally initialized for FedPer and FedRep). We note the structural trade-off in advance: in our architecture, parameter-averaging methods upload 411–535 KiB per client per round, an order of magnitude more than the 41.6 KiB parameter-free payload of FedMCP++ (Section 5.1), so accuracy comparisons should be read jointly with the communication budget.

Table 10 reports the comparison under uniform partitioning and Table 11 repeats it under Dirichlet (β = 0.3) label skew, with client-level accuracy under both protocols of Section 3.4; in Table 11 the deployment-distribution protocol is listed first, since it is the metric under which personalization can manifest. Baseline entries and the whole of Table 11 use seed 0 with the released implementation; the FedMCP++ entries of Table 10 report mean ± standard deviation over the five seeds of Section 5.2(e) (mean only for Worst and Gap); the corresponding macro-F1 values are provided in the released per-run files (under label skew, per-client macro-F1 diverges from accuracy, as expected for specialized models).

Three regularities organize Table 11 and Table 12. First, no method dominates: the ranking depends jointly on the partitioning regime and on the client-accuracy protocol, which is precisely why Section 3.4 reports both. Under uniform partitioning FedAvg attains the best full-test client accuracy in 17 of 18 configurations and the best global accuracy in 13, while under label skew FedRep attains the best deployment-distribution accuracy in 16 of 18. Second, FedMCP++ occupies the robust middle of the consensus–specialization spectrum at one tenth of the uplink: it exceeds FedPer in global accuracy in 14 of 18 uniform configurations and leads the personalized family on the full-test protocol in the scarce STL-10 cells of both panels; under skew it exceeds the consensus model on the deployment-distribution protocol across the harder RGB benchmarks (by +4.1 to +11.3 pp on CIFAR-10 and +16.8 to +22.1 pp on STL-10), while trailing it on the easier grayscale ones; and it is the only method that never degrades to chance level in any of the 36 cells. Third, both endpoints of the spectrum exhibit failure modes: full-model averaging collapses to chance on SVHN under label skew at 20 and 30 clients (global accuracy 0.160 and 0.159; a single-seed observation, to be revisited with additional seeds), and the purely local heads of FedPer and FedRep collapse in the data-scarcest uniform STL-10 cells (client accuracy 0.20–0.23 versus 0.25–0.28 for FedMCP++). FedMCP++’s own weakest regime is also visible and reported: its global accuracy on SVHN degrades markedly under skew (0.71 → 0.22 from the uniform to the Dirichlet 30-client cell), indicating that the proxy-mediated signal is noisier than parameter averaging when local label distributions are severely skewed. FedPer had not plateaued at the fixed 15-round budget in several cells; the budget is applied identically to all methods. Five-seed dispersion (Section 5.2(e)) supplies the scale for reading these margins: standard deviations of global accuracy are at most 0.23 pp on the grayscale benchmarks and 0.3–1.6 pp on the RGB ones, so the family margins in the scarce STL-10 cells and the RGB deployment-distribution gaps exceed that scale several-fold, while sub-0.2 pp differences should be read as parity.

## 5. Discussion

In this paper, we have introduced FedMCP++, a modular federated learning framework that integrates private expert heads, per-client backbones aligned by distillation, contrastive alignment, and prototype-guided aggregation, under a communication protocol that exchanges no model parameters. Our empirical evaluation spans six vision benchmarks (MNIST, Fashion-MNIST, SVHN, KMNIST, STL-10, CIFAR-10), three client-scale regimes (10, 20, and 30 clients), two knowledge-based baselines (Local and EnsKD), and three ablations of the method. Read jointly, Table 7, Table 8 and Table 9 do not support a claim of uniform superiority; they support a more differentiated conclusion. Parameter-free collaboration is most valuable exactly where personalization matters most—under severe per-client data scarcity—raising client-level accuracy over independent local training in twelve of the eighteen configurations, with margins that grow with the federation size on the grayscale benchmarks and on STL-10. Conversely, on SVHN and CIFAR-10 at the studied budgets, the independent-training ensemble remains the strongest global model. Crucially, whichever configuration is preferred, the backbone budget is identical throughout (Table 6), and the communication budget of the collaborative variants is fixed and small (Section 5.1).

A central architectural property is that the private expert heads let each client specialize on its local distribution while the backbones remain semantically coupled through the proxy-set signals. Because every model component is private, the framework cannot suffer the parameter-interference failure modes of monolithic averaging; the price is that global coherence must be earned through the distillation signal alone. The results quantify both sides of this trade: coupling through soft predictions is sufficient to lift client-level accuracy where local data are too scarce for independent training (e.g., +2.74 pp CAcc on KMNIST with 30 clients), yet the same coupling can drag clients toward a weak consensus on SVHN and CIFAR-10 at high client counts, where the intermediate teachers are poor.

Our ablation studies clarify the role of each component, provided they are read against the run-to-run noise envelope of Section 5.2. The replicate rows (formerly “w/o heads”) are duplicate executions of the identical configuration and bound that envelope at up to 0.73 pp GAcc. Within it, the contrastive term is close to cost-neutral at all scales—its largest effects (≈±0.5 pp) do not clearly exceed noise—so its justification is at present architectural (a dimension-matched alignment of embeddings to teacher beliefs) rather than empirical. Prototype alignment, by contrast, produces effects that do exceed the envelope, in both directions: it contributes up to +1.37 pp GAcc and +1.86 pp CAcc on KMNIST at 20 clients, and costs up to −3.24 pp GAcc on STL-10 at 30 clients. We also correct an argument made in the original submission: because the ablated variants are not parameter-averaging methods, none of them is structurally analogous to FedRep or FedPer, and no indirect conclusion about representation-decoupling baselines can be drawn from them; the direct comparison is instead provided in Section 4.4.

The configurations in which Local or EnsKD outperforms the full model deserve a mechanistic reading. First, on SVHN and CIFAR-10 the per-client shards are comparatively large, so independent models are individually strong and their ensemble is a high bar; distilling toward any consensus—weighted or not—helps only if the consensus is better than what each client already knows. Second, distillation operates on a 1000-sample proxy: on visually rich RGB data this is a narrow window through which to transfer knowledge, whereas on the grayscale tasks it covers the input manifold far better. Third, the accuracy-weighted teacher demonstrably helps exactly where teacher quality is heterogeneous (SVHN at 20 clients: +1.34 pp GAcc over the uniform-average EnsKD), which is consistent with this reading.

Beyond averages, fairness claims require client-level dispersion statistics, and the original submission asserted them (“over 30% lower standard deviation”, “up to 40% narrower best-to-worst gap”) without having logged per-client metrics; we retract those specific figures. The revised protocol of Section 3.4 records the standard deviation of client accuracies, the worst-client accuracy, and the best-to-worst gap for every configuration, and the released implementation writes them to per-run files. What can already be stated from the design is that, under heterogeneous client distributions, the private heads remove the mechanism by which a single global parameterization “washes out” minority clients. Under (near-)IID partitioning, by contrast, no minority distributions exist for that mechanism to protect, and a single well-fitted global model can itself exhibit low client-level dispersion; whether the design translates into measurably lower dispersion is therefore a regime-dependent empirical question, which the new statistics document separately for the uniform and Dirichlet partitionings. The completed grid supports the conditioned mechanism against consensus models: at Dirichlet (β = 0.3) on STL-10 with 30 clients, the worst client of FedMCP++ attains 0.302 accuracy on its own label distribution versus 0.096 under the global-averaging model (Table 11); within the personalized family, the worst-client statistics of FedMCP++, FedPer, and FedRep are at parity in that cell (0.302/0.310/0.304).

Table 12 reports the client-level fairness statistics of FedMCP++ against independent local training across all eighteen uniform configurations.

Relative to independent training, FedMCP++ improves the worst-performing client in 12 of the 18 configurations and narrows the best-to-worst gap in 13, with the largest worst-client gains in the data-scarce cells; its mean client accuracy exceeds independent training in 13 of 18 under the five-seed statistics. The improvements are modest in the data-rich grayscale cells and concentrate where collaboration matters, consistent with the scarcity mechanism of Section 4.4; the corresponding statistics under label skew appear in Table 11.

From a practical standpoint, the communication properties are the clearest asset, and we correct the original description here: the protocol involves no model upload or download at all. Each client uploads, per round, a fixed-size message of 42.6 KB—the 1000 × 10 matrix of softened proxy predictions plus the 10 × 64 prototype matrix in 32-bit precision—and downloads a message of the same dimensions; nothing about this payload depends on the backbone size, the head size, or the number of clients. Relative to synchronizing the full model every round (544.9 KB on the RGB benchmarks, 420.9 KB on the grayscale ones), this is a 12.8× and 9.9× per-round reduction, respectively, and the factor grows linearly with model capacity. The trade-offs are equally explicit: the server holds no model (it only averages predictions), while ensemble-level global inference, if desired at deployment time, requires querying multiple clients (Section 3.1.6).

In sum, FedMCP++ is best characterized not as a method that dominates its baselines, but as a modular, parameter-free collaboration layer whose components can be enabled where they help: the full configuration on data-scarce grayscale tasks, prototype alignment disabled on data-scarce RGB tasks, and plain local training retained where per-client data suffice. The modularity is not rhetorical—each component is a documented switch in the released implementation—and the fixed, model-independent communication footprint holds in every configuration.

**Figure 3:**
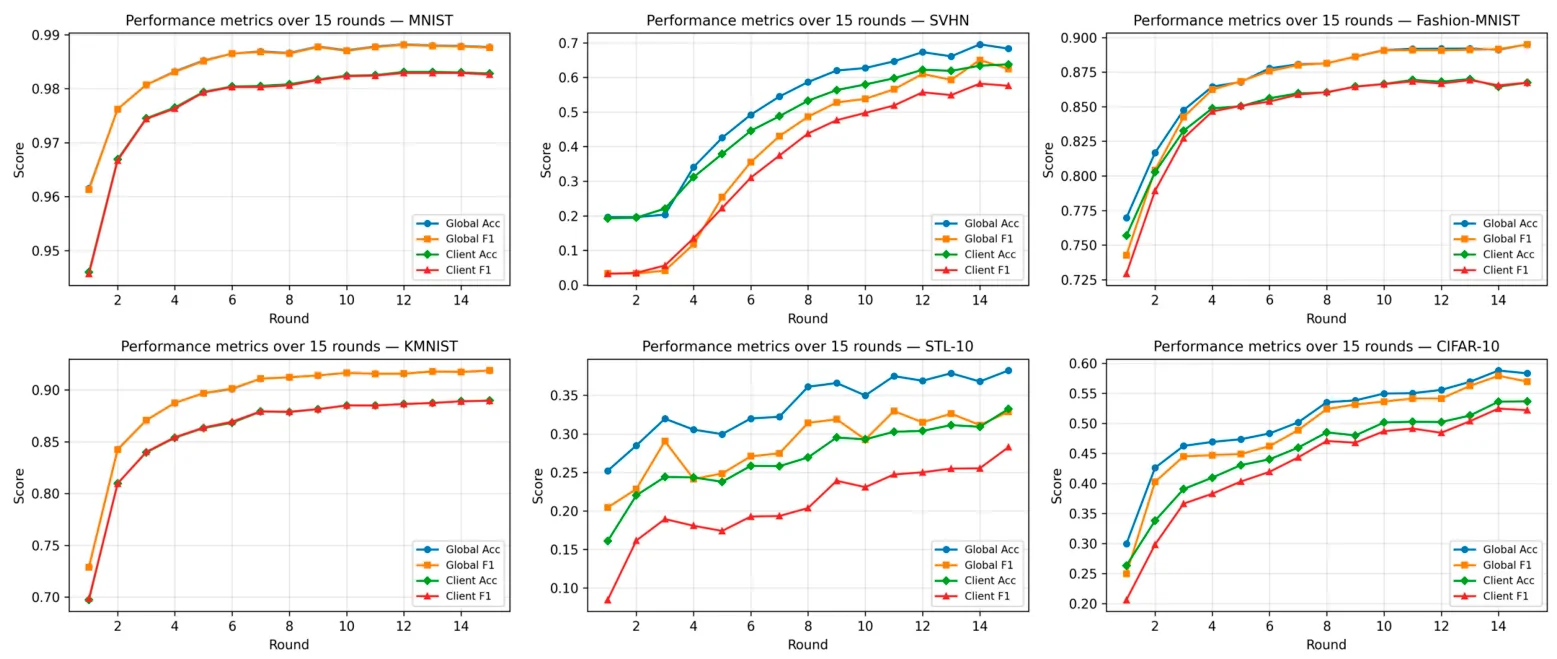
Per-round performance of the full FedMCP++ configuration on the six benchmarks with 10 clients: global and client-level accuracy and macro-F1 over the 15 communication rounds.

As illustrated in Figure 3, the per-round trajectories of the full FedMCP++ configuration are shown for the six benchmark datasets—MNIST, SVHN, Fashion-MNIST, KMNIST, STL-10, and CIFAR-10—in the 10-client setting. Each subfigure tracks four metrics—global accuracy, global macro-F1, client accuracy, and client macro-F1—over the 15 communication rounds. The grayscale benchmarks converge within the first three to five rounds and then plateau, whereas SVHN, CIFAR-10, and especially STL-10 improve more slowly and exhibit visible round-to-round oscillation, reflecting their higher visual complexity and the small per-client sample counts.

The gap between the global and client-level curves narrows over the rounds on the grayscale tasks, consistent with the distillation and prototype signals pulling individual clients toward the ensemble. On CIFAR-10 and STL-10, the curves saturate at lower levels and the global–local gap persists, indicating that with 500–5000 samples per client the proxy-mediated signal cannot fully compensate for data scarcity on visually diverse RGB data. These round-wise plots are descriptive of the full configuration only; comparative statements across methods are confined to Table 7, Table 8 and Table 9.

A closer inspection of Figure 3 also explains the larger round-to-round fluctuations on SVHN and STL-10. Both datasets combine severe per-client data scarcity with substantial intra-class visual ambiguity (low-resolution street-view digits and few-shot natural images, respectively). In the early rounds, client embeddings on the public proxy set are not yet aligned, so the aggregated teacher predictions and prototypes are comparatively noisy and can momentarily pull individual clients in conflicting directions; as the contrastive alignment matures, the shared feature space stabilizes and the oscillations visibly dampen. Regarding scale, prototype aggregation is a fixed-size averaging operation over class-wise centroids: its output dimensionality is independent of the number of participating clients, and—under standard independence assumptions—the variance of the averaged teacher prototypes decreases on the order of 1/N as the federation grows, consistent with the absence of divergence when scaling from 10 to 30 clients in Table 7, Table 8 and Table 9. Statistical reliability across random seeds is addressed by the replicate rows and the multi-seed protocol of Section 5.2.

### 5.1. Runtime and Efficiency Considerations

In addition to accuracy, the framework offers a transparent computational profile. As Table 13 shows, the full FedMCP++ pipeline increases end-to-end training time over the communication-free Local baseline by 12–52% depending on the dataset (e.g., from 300.3 s to 378.7 s on MNIST and from 748.2 s to 933.6 s on CIFAR-10), with the overhead dominated by the additional forward/backward passes of the distillation phase on the proxy set.

The component-level timings separate these costs. Disabling the contrastive term changes total runtime by at most 3%—within measurement noise, and not consistently in one direction—confirming that the projection and contrastive-loss computations are lightweight. Removing prototype alignment shortens runtime by up to 6.7% (e.g., from 1213.7 s to 1132.9 s on STL-10), making it the most expensive optional component. The distillation-only EnsKD baseline runs 5–21% slower than Local and 7–20% faster than the full model, bracketing the cost of the additional alignment machinery.

Across all six vision tasks, even those with high resolution or significant heterogeneity (SVHN, STL-10, CIFAR-10), the total training time for fifteen federated rounds remains comfortably under 30 min with 10 clients. These measurements show that the behaviors documented earlier are obtained with only a modest computational burden. Consequently, the framework remains straightforward to run on commodity hardware. The memory footprint of the personalization machinery is similarly modest: as quantified in Section 4.2 and Table 6, the client-resident modules (the private expert head and the two projection layers) account for less than 2% of the total model parameters, which corresponds to only a few kilobytes of additional on-device storage in 32-bit floating-point precision—negligible relative to the backbone itself on any contemporary edge device.

**Figure 4:**
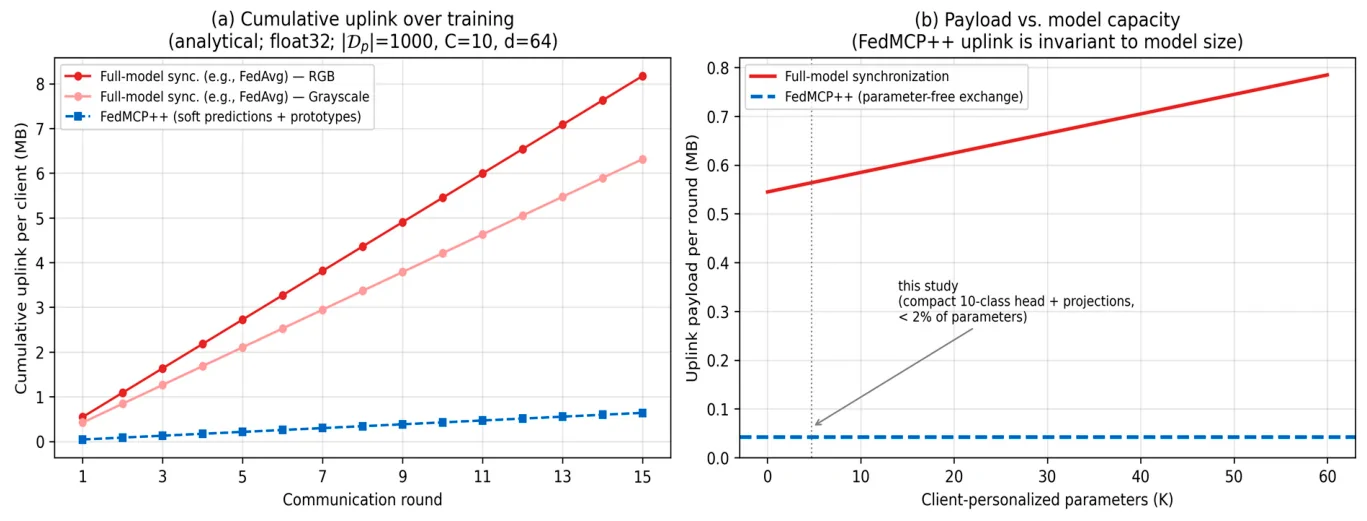
Analytical communication profile of the implemented FedMCP++ protocol under 32-bit floating-point transmission. (**a**) Cumulative per-client uplink over the 15 federated rounds: full-model synchronization (544.9/420.9 KB per round on the RGB/grayscale families) versus the parameter-free FedMCP++ exchange (42.6 KB per round of proxy predictions and prototypes). (**b**) Per-round uplink payload as a function of the capacity allocated to client-personalized modules: synchronization-based payloads grow linearly with the number of transmitted parameters, whereas the FedMCP++ payload is constant, fixed by the proxy-set size and the number of classes.

Beyond runtime and memory, communication cost is the third axis of deployment efficiency, and the audited protocol makes it the framework’s strongest property. Figure 4 reports the analytical communication profile of the implemented protocol under 32-bit floating-point transmission. Panel (a) compares cumulative per-client uplink over the 15 training rounds: full-model synchronization transmits 544.9 KB per round on the RGB benchmarks (SVHN, STL-10, CIFAR-10) and 420.9 KB on the grayscale ones, whereas FedMCP++ transmits no parameters at all—its 42.6 KB payload consists of the 1000 × 10 matrix of softened proxy predictions plus the 10 × 64 prototype matrix—yielding 12.8× and 9.9× per-round reductions, respectively. Panel (b) shows the consequence of parameter-freedom: the payload of synchronization-based methods grows linearly with the capacity allocated to the model, while the FedMCP++ payload is a horizontal line fixed by the proxy size and the class count, independent of backbone, head, and client count. The downlink mirrors the uplink, as the broadcast teacher has the same dimensions. Because every method is trained for the same fixed budget of 15 rounds, the accuracy trajectories of Figure 3 and the cost profile of Figure 4 can be read jointly: the collaborative behaviors of Table 7, Table 8 and Table 9 are obtained at roughly 8–10% of the communication cost of parameter-synchronization approaches.

### 5.2. Limitations and Revision Experiments

The audit performed for this revision surfaced limitations that we make explicit here, together with the concrete experiments—implemented in the released fedmcp_v2 code—that address them.

(a) **Partitioning**: The main tables use uniform random partitioning; heterogeneity arises from per-client sample scarcity rather than engineered label skew (Section 4.1). The implementation supports Dirichlet(β) label-skew partitioning [46]. A first completed sweep on Fashion-MNIST (10 clients, seed 0) characterizes the sensitivity concretely: the global-accuracy gap between FedAvg and FedMCP++ grows monotonically with skew severity (0.1 pp under uniform partitioning, 0.7 pp at β = 0.5, 2.9 pp at β = 0.3, and 16.4 pp at β = 0.1), while the two client-accuracy protocols of Section 3.4 diverge sharply—at β = 0.1 the consensus model leads on the full test split, whereas on each client’s own label distribution FedMCP++ attains 0.939 mean client accuracy versus 0.854 for FedAvg (+8.4 pp; worst client +11.3 pp); the local-protocol advantage persists at β = 0.3 (+1.9 pp; worst client +3.2 pp) and closes at β = 0.5 (+0.3 pp), so the personalization premium grows monotonically with skew severity when measured on deployment distributions. The full-grid Dirichlet(β = 0.3) comparison across all six benchmarks and three federation sizes is now reported in Table 11.

Table 14 extends the label-skew analysis to β ∈ {0.1, 0.5} for FedMCP++, FedAvg, and independent local training at 10 clients, complementing the β = 0.3 grid of Table 11; the deployment-distribution protocol is again listed first.

Three observations follow. First, the deployment-distribution advantage of FedMCP++ over the consensus model grows with skew severity on the harder benchmarks: at β = 0.1 it reaches +9.1 pp on Fashion-MNIST, +4.1 pp on KMNIST, +7.6 pp on CIFAR-10, and +42.9 pp on STL-10, with MNIST—at a 0.98 ceiling—the exception; the SVHN margin (+44.6 pp) is dominated by the collapse of full-model averaging there (deployment-distribution accuracy 0.102) and is therefore not credited as a like-for-like win. Second, the local-protocol accuracy of FedMCP++ itself rises as skew sharpens on four of the six benchmarks (on STL-10, 0.457 → 0.516 → 0.675 from β = 0.5 to 0.1): narrower client distributions are easier for a personalized model to master. Third—and this is the honest boundary of the approach—at extreme skew, independent local training becomes the strongest own-distribution model on all six benchmarks, and on the RGB benchmarks it also matches or exceeds the ensemble global accuracy of FedMCP++ (0.492 versus 0.355 on CIFAR-10): under near-disjoint client distributions, proxy-mediated collaboration is counterproductive relative to not collaborating at all, and the practical recommendation is to fall back to independent training in that regime. The β = 0.3 grid of Table 11 marks where collaboration re-emerges as beneficial.

(b) **Scale**: The largest federation studied is 30 clients; the implementation runs arbitrary client counts at unchanged communication cost per client. The 100-client experiment covers five of the six benchmarks; STL-10 is excluded by design, since its 5000 training images would leave 50 samples per client, beneath any meaningful local training.

Table 15 reports the 100-client experiment for FedMCP++ and independent local training.

Three observations follow. First, the client-level benefit of proxy-mediated collaboration persists at contemporary scale and is concentrated exactly where the paper claims it: FedMCP++ exceeds independent training in mean client accuracy on four of the five benchmarks (+0.5 pp on MNIST, +2.2 pp on KMNIST, +1.5 pp on CIFAR-10, +0.1 pp on SVHN) and in worst-client accuracy on the same four (up to +1.7 pp), with Fashion-MNIST the honest exception (−0.5 pp). Second, the ensemble of one hundred independent models is slightly stronger on the global protocol (−0.3 to −1.6 pp for FedMCP++): distillation trades ensemble diversity for client-level alignment, consistent with the consensus–specialization reading of Section 4.4. Third, SVHN at 100 clients is a wall for both methods—both sit at the 0.196 majority-class level—because 732 samples over 15 rounds do not suffice to learn SVHN at all; the failure is budget-induced and method-independent, and the cell is reported for completeness. The per-client communication payload is scale-invariant by construction, so the 41.6 KiB budget holds unchanged at this federation size.

(c) **Proxy sensitivity:** All main results use a 1000-sample in-domain proxy that overlaps the evaluation split by 10%. The implementation exposes the proxy size, its class composition, an evaluation-disjoint mode, and cross-domain proxies.

Table 16 reports the proxy-sensitivity analysis on Fashion-MNIST and CIFAR-10.

Two observations follow. First, the method is robust to the proxy budget: across an eightfold change in proxy size (250 to 2000 samples), global accuracy varies by only 0.25 pp on Fashion-MNIST and 1.02 pp on CIFAR-10—within the respective single-seed noise envelopes—and a proxy of 250 public samples, i.e., 25 per class, already suffices; client-level accuracy shows a mild monotone gain with proxy size (+0.9 pp and +1.0 pp from the smallest to the largest budget), a diminishing return rather than a dependence. Second, and directly addressing the overlap concern: with a proxy fully disjoint from the evaluation split, global and client-level accuracies change by at most 0.14 pp relative to the overlapping protocol of the main tables—the overlap conferred no measurable advantage, so the results of Table 7, Table 8, Table 9, Table 10 and Table 11 are not inflated by proxy–evaluation contamination.

(d) **Privacy quantification**: Section 3.1.7 argues the structural elimination of gradient- and parameter-based leakage; the residual output channel is quantified with a confidence-based membership-inference evaluation [44,45], executed identically for every method in the revision runs. The complete measurement across all six benchmarks follows.

Table 17 reports the attack AUC for FedAvg, independent local training, and FedMCP++ on every benchmark.

Three observations follow. First, the ordering FedAvg < FedMCP++ < Local holds on five of the six benchmarks (on Fashion-MNIST, FedMCP++ and Local are indistinguishable at 0.5510 versus 0.5503): distillation acts as a mild regularizer that reduces output-channel leakage relative to purely local training, while the still-lower AUC of FedAvg is obtained at the cost of transmitting the full parameter vector every round—precisely the channel that gradient-inversion attacks exploit and that FedMCP++ eliminates. Second, leakage through the output channel is dataset-dependent and is a real, measurable risk on KMNIST (AUC 0.715 for Local and 0.704 for FedMCP++): models that fit an easy benchmark nearly perfectly become confident on their members, and the residual channel of Section 3.1.7 is then not negligible; this is exactly the case for which the output-side mitigations discussed there (temperature, perturbation, or restricting proxy exposure) are intended. Third, on the harder RGB benchmarks the AUC is at or marginally below 0.5 for every method: within the 15-round budget the models do not memorize their training shards enough for the confidence attack to find signal, and values slightly below 0.5 reflect attack noise rather than any protective inversion.

(e) **Statistical reliability**: In the originally submitted version, all main-table numbers stemmed from a single seeded run (seed 0), with same-seed replicate rows bounding run-to-run variation at up to 0.73 pp GAcc (SVHN), 0.49 pp (STL-10), 0.42 pp (CIFAR-10), and at most 0.10 pp on the grayscale benchmarks. The five-seed statistics below supersede that replicate-based reading: differences are now judged against the across-seed dispersion. Across the completed five-seed grid, the across-seed standard deviation of global accuracy is at most 0.23 pp on the grayscale benchmarks (median 0.11 pp) and 0.3–1.6 pp on the RGB benchmarks (largest on STL-10), modestly exceeding the same-seed replicate envelope, as anticipated. The released grid driver executes all five seeds of every knowledge-transfer method on a single machine (phases 3 and 3b), so the reported dispersion is not confounded by hardware differences; the FedMCP++ column of Table 10 carries the same five-seed dispersion. As an independent check, the complete seed-0 grid of the FedMCP++, w/o contrastive, w/o protos, and EnsKD rows was re-executed on a second machine and agrees with the published values of Table 7, Table 8 and Table 9 within mean absolute deviations of 0.1–0.3 pp on the grayscale benchmarks and 1.0–1.3 pp on the RGB benchmarks.

Table 7, Table 8 and Table 9 now report mean ± standard deviation over five independent seeds for every method row (FedMCP++, w/o contrastive, w/o protos, Local, and EnsKD), all computed on a single machine; the replicate rows are retained as same-seed re-runs of the original protocol.

*Future directions* include extending FedMCP++ to multimodal and temporal data settings, incorporating secure aggregation techniques for enhanced privacy guarantees, combining the parameter-free exchange with differential-privacy noise on the transmitted predictions, and exploring dynamic head sharing for adaptive expert routing in massively distributed environments. The comparison against representation-decoupling baselines, the proxy-sensitivity analysis, and the multi-seed evaluation are now part of the study (Section 4.4 and Section 5.2). Remaining empirical extensions include seed-level statistics for the Dirichlet panel and for the parameter-averaging baselines (the released driver exposes the latter as phase 3c), and proxy construction under domain shift between the proxy and client distributions, for which the implementation already provides a cross-dataset proxy switch.

## 6. Conclusions

This study introduced FedMCP++, a modular and communication-efficient personalized federated learning framework in which clients own complete private models and collaborate exclusively through soft predictions and class prototypes computed on a small public proxy set. The design directly addresses communication bottlenecks—its per-round payload is fixed at 42.6 KB, independent of model capacity—and structurally eliminates parameter- and gradient-based privacy attack surfaces, properties suited to edge and IoT deployments.

Across six vision benchmarks and three federation sizes, the audited results support a differentiated conclusion rather than uniform superiority. Parameter-free collaboration raises client-level accuracy over independent local training in twelve of eighteen configurations, with the largest margins under severe per-client data scarcity (up to +2.7 pp on KMNIST and +2.4 pp on STL-10 at 20–30 clients), and the accuracy-weighted teacher outperforms uniform ensemble distillation most visibly on SVHN. Independent training ensembles remain the strongest global models on SVHN and CIFAR-10 at the studied budgets, and prototype alignment proves regime-dependent—beneficial on grayscale tasks at moderate scale, counter-productive in the most data-scarce RGB settings. All configurations share an identical backbone budget, so these differences are attributable to the collaboration mechanisms themselves.

In summary, FedMCP++ offers a transparent and practical collaboration layer for personalized federated learning: every component is a documented switch in the released implementation, every exchanged byte is accounted for, and the regimes in which each mechanism helps are identified empirically. We believe this makes the framework an honest foundation for bandwidth- and privacy-constrained federated deployments, with the verification programme of Section 5.2 now executed in full, completing the picture on non-IID partitioning, scale, proxy sensitivity, privacy, and statistical reliability (Table 10, Table 11, Table 12, Table 13, Table 14, Table 15, Table 16 and Table 17).

## Figures and Tables

**Table 1:** Comprehensive comparison of federated and personalized FL methods.

Method	Personalization Strategy	Communication Efficiency	Privacy Mechanism	Key Limitation
FedAvg [35]	None	Full-model synchronization	Parameter aggregation	Poor under non-IID data
FedProx [8]	Proximal regularization	Full-model synchronization	Proximal stabilization	Limited explicit personalization
FedMA [36]	Layer-wise weight matching	Hierarchical aggregation	Weight alignment	Computational complexity
MOON [15]	Model-level contrastive learning	Full-model synchronization	Contrastive regularization	Limited client-specific flexibility
FedPer [7]	Personalized client-specific layers	Shared backbone only	Local layers privacy	Lacks global–local alignment
FedRep [6]	Global representation, personal heads	Shared backbone only	Local classifier training	Limited alignment of embeddings
FedSeq [40]	Sequential expansion of layers	Layer-wise synchronization	Decoupled local training	Complex scheduling
pFedBayes [37]	Variational Bayesian inference	Probabilistic synchronization	Bayesian parameter privacy	High computational cost
pFedLA [38]	Personalized layer aggregation	Layer-wise synchronization	Local weighted aggregation	Hyperparameter sensitivity
FedDWA [39]	Dynamic weighted aggregation	Partial synchronization	Client similarity weighting	Increased server complexity
FedAF [28]	Synthetic dataset sharing	Synthetic data exchange	Synthetic data privacy	Quality of synthetic data critical
PEFT [29]	Prompt-based fine-tuning	Prompt parameter sync	Prompt-level privacy	Depends on large pre-trained models
HyFDCA [41]	Hybrid data/model splits	Dual averaging	Convex optimization privacy	Primarily for convex optimization
FairDPFL-SCS [42]	Dynamic learning rate + strategic selection	Model updates only	Decentralized model updates	Strategic selection complexity
**FedMCP++ (ours)**	Contrastive distillation + expert heads	Fixed-size logit + prototype messages (no parameter exchange)	Proxy-based contrastive alignment	Requires small public dataset

**Table 2:** Comparison of different optimizers. Metrics include global accuracy (GAcc), global F1-score (GF1), client accuracy (CAcc), client F1-score (CF1), and total training time.

Optimizer	GAcc	GF1	CAcc	CF1	Time (s)
SGD	0.9882	0.9881	0.9828	0.9827	138.3
Adam	0.9875	0.9875	0.9767	0.9766	142.6
RMSprop	0.9845	0.9844	0.9691	0.9689	139.3
Adagrad	0.9860	0.9859	0.9810	0.9809	138.7
AdamW	0.9855	0.9854	0.9724	0.9723	140.5

**Table 3:** Comparison of different backbone architectures using the SGD optimizer.

Backbone	GAcc	GF1	CAcc	CF1	Time (s)
small	0.9882	0.9881	0.9830	0.9829	135.4
resnet18	0.9891	0.9890	0.9727	0.9728	223.8
densenet121	0.9930	0.9929	0.9899	0.9898	844.4
mobilenet_v2	0.9904	0.9903	0.9809	0.9808	353.6

**Table 4:** Hyperparameter configuration used in all experiments.

Parameter	Value
Number of Clients	10, 20, 30
Rounds	15
Local Epochs	1
Batch Size	64
Backbone Learning Rate	0.05
Expert Head Learning Rate	0.05
Momentum	0.9
Gradient Clipping	5.0
KL Temperature	1.5
Latent Dim (Backbone Output)	64
Projection Dim (Contrastive)	32
Contrastive Loss Weight (λ)	0.1
Prototype Loss Weight (α)	0.1
Contrastive Temperature (τ)	1.5
Public Proxy-Set Size |Dp|	1000 samples
Optimizer	SGD, momentum 0.9
Client Participation	Full (all clients, every round)
Data Partitioning	Uniform random, equal disjoint shards
Random Seed	0 (PyTorch, NumPy, Python)

**Table 5:** Summary of datasets used in our experiments, including domain, number of classes, image size, color type, and train/test splits. STL-10 images are resized to 32 × 32 pixels in all experiments.

Dataset	Domain	Classes	Image Size	Color	Train/Test
MNIST	Handwritten Digits	10	28 × 28	Grayscale	60,000/10,000
Fashion-MNIST	Fashion Articles	10	28 × 28	Grayscale	60,000/10,000
KMNIST	Japanese Characters	10	28 × 28	Grayscale	60,000/10,000
SVHN	Street View House Numbers	10	32 × 32	RGB	73,257/26,032
CIFAR-10	Natural Objects	10	32 × 32	RGB	50,000/10,000
STL-10	Large-scale Visual Objects	10	96 × 96 (resized to 32 × 32)	RGB	5000/8000

**Table 6:** Model complexity summary by dataset (in thousands).

Dataset	FLOPsk	Paramsk
MNIST	1116.42	105.22
Fashion-MNIST	1116.42	105.22
KMNIST	1116.42	105.22
SVHN	1753.09	136.22
STL-10	1753.09	136.22
CIFAR-10	1753.09	136.22

**Table 7:** The obtained findings on six vision datasets with 10 clients. Metrics shown: global accuracy (GAcc), global F1 (GF1), client-local accuracy (CAcc), client F1 (CF1).

Dataset	Variant	GAcc	GF1	CAcc	CF1
MNIST	FedMCP++	0.9884 ± 0.0004	0.9883 ± 0.0004	0.9836 ± 0.0003	0.9835 ± 0.0004
	replicate	0.9877	0.9876	0.9828	0.9826
	w/o contrastive	0.9884 ± 0.0006	0.9883 ± 0.0006	0.9836 ± 0.0003	0.9835 ± 0.0003
	w/o protos	0.9884 ± 0.0005	0.9884 ± 0.0005	0.9837 ± 0.0004	0.9836 ± 0.0004
	Local	0.9884 ± 0.0008	0.9883 ± 0.0008	0.9822 ± 0.0005	0.9821 ± 0.0005
	EnsKD	0.9884 ± 0.0006	0.9884 ± 0.0006	0.9838 ± 0.0003	0.9836 ± 0.0003
SVHN	FedMCP++	0.6976 ± 0.0078	0.6465 ± 0.0147	0.6486 ± 0.0065	0.5944 ± 0.0121
	replicate	0.6906	0.6346	0.6466	0.5879
	w/o contrastive	0.6987 ± 0.0061	0.6483 ± 0.0107	0.6454 ± 0.0052	0.5922 ± 0.0091
	w/o protos	0.6968 ± 0.0064	0.6443 ± 0.0086	0.6476 ± 0.0070	0.5924 ± 0.0106
	Local	0.7014 ± 0.0019	0.6529 ± 0.0037	0.6485 ± 0.0057	0.5960 ± 0.0063
	EnsKD	0.6994 ± 0.0078	0.6493 ± 0.0123	0.6463 ± 0.0061	0.5929 ± 0.0118
Fashion-MNIST	FedMCP++	0.8956 ± 0.0023	0.8949 ± 0.0020	0.8697 ± 0.0016	0.8691 ± 0.0012
	replicate	0.8948	0.8948	0.8701	0.8701
	w/o contrastive	0.8957 ± 0.0017	0.8950 ± 0.0017	0.8698 ± 0.0013	0.8691 ± 0.0015
	w/o protos	0.8959 ± 0.0011	0.8950 ± 0.0011	0.8710 ± 0.0014	0.8702 ± 0.0016
	Local	0.8961 ± 0.0014	0.8952 ± 0.0014	0.8641 ± 0.0029	0.8633 ± 0.0032
	EnsKD	0.8964 ± 0.0014	0.8957 ± 0.0015	0.8707 ± 0.0013	0.8700 ± 0.0010
KMNIST	FedMCP++	0.9183 ± 0.0004	0.9182 ± 0.0004	0.8909 ± 0.0012	0.8908 ± 0.0012
	replicate	0.9186	0.9185	0.8901	0.8900
	w/o contrastive	0.9183 ± 0.0008	0.9182 ± 0.0008	0.8908 ± 0.0012	0.8908 ± 0.0012
	w/o protos	0.9182 ± 0.0009	0.9180 ± 0.0009	0.8909 ± 0.0013	0.8908 ± 0.0013
	Local	0.9151 ± 0.0008	0.9149 ± 0.0008	0.8759 ± 0.0010	0.8758 ± 0.0010
	EnsKD	0.9181 ± 0.0009	0.9179 ± 0.0009	0.8908 ± 0.0010	0.8907 ± 0.0010
STL-10	FedMCP++	0.3894 ± 0.0063	0.3445 ± 0.0096	0.3296 ± 0.0081	0.2821 ± 0.0076
	replicate	0.3871	0.3320	0.3310	0.2809
	w/o contrastive	0.3881 ± 0.0056	0.3432 ± 0.0112	0.3273 ± 0.0038	0.2791 ± 0.0071
	w/o protos	0.3882 ± 0.0079	0.3440 ± 0.0130	0.3337 ± 0.0034	0.2852 ± 0.0058
	Local	0.3843 ± 0.0065	0.3357 ± 0.0078	0.3177 ± 0.0072	0.2650 ± 0.0097
	EnsKD	0.3889 ± 0.0077	0.3464 ± 0.0109	0.3340 ± 0.0035	0.2852 ± 0.0037
CIFAR-10	FedMCP++	0.5832 ± 0.0067	0.5720 ± 0.0071	0.5289 ± 0.0075	0.5157 ± 0.0067
	replicate	0.5875	0.5755	0.5346	0.5224
	w/o contrastive	0.5835 ± 0.0073	0.5720 ± 0.0074	0.5290 ± 0.0082	0.5156 ± 0.0076
	w/o protos	0.5847 ± 0.0042	0.5730 ± 0.0056	0.5304 ± 0.0037	0.5172 ± 0.0028
	Local	0.5975 ± 0.0039	0.5872 ± 0.0039	0.5439 ± 0.0025	0.5329 ± 0.0032
	EnsKD	0.5849 ± 0.0057	0.5723 ± 0.0072	0.5303 ± 0.0052	0.5162 ± 0.0051

Note: method rows report five-seed mean ± standard deviation; replicate rows are same-seed re-runs of the original protocol (Section 5.2(e)).

**Table 8:** The obtained findings on six vision datasets with 20 clients. Metrics shown: global accuracy (GAcc), global F1 (GF1), client-local accuracy (CAcc), client F1 (CF1).

Dataset	Variant	GAcc	GF1	CAcc	CF1
MNIST	FedMCP++	0.9849 ± 0.0004	0.9849 ± 0.0004	0.9786 ± 0.0003	0.9785 ± 0.0003
	replicate	0.9849	0.9848	0.9782	0.9781
	w/o contrastive	0.9849 ± 0.0005	0.9848 ± 0.0005	0.9786 ± 0.0004	0.9785 ± 0.0004
	w/o protos	0.9849 ± 0.0005	0.9848 ± 0.0005	0.9787 ± 0.0003	0.9785 ± 0.0003
	Local	0.9849 ± 0.0005	0.9848 ± 0.0005	0.9762 ± 0.0003	0.9761 ± 0.0003
	EnsKD	0.9849 ± 0.0004	0.9848 ± 0.0004	0.9787 ± 0.0004	0.9785 ± 0.0004
SVHN	FedMCP++	0.5570 ± 0.0083	0.4416 ± 0.0101	0.5019 ± 0.0074	0.3923 ± 0.0100
	replicate	0.5792	0.4678	0.5156	0.4127
	w/o contrastive	0.5549 ± 0.0098	0.4395 ± 0.0127	0.4998 ± 0.0084	0.3909 ± 0.0124
	w/o protos	0.5597 ± 0.0158	0.4471 ± 0.0218	0.5044 ± 0.0132	0.3978 ± 0.0181
	Local	0.5693 ± 0.0106	0.4511 ± 0.0140	0.5158 ± 0.0117	0.4065 ± 0.0145
	EnsKD	0.5563 ± 0.0154	0.4426 ± 0.0200	0.5021 ± 0.0108	0.3940 ± 0.0147
Fashion-MNIST	FedMCP++	0.8841 ± 0.0010	0.8839 ± 0.0010	0.8543 ± 0.0006	0.8543 ± 0.0006
	replicate	0.8842	0.8838	0.8548	0.8546
	w/o contrastive	0.8842 ± 0.0012	0.8840 ± 0.0013	0.8542 ± 0.0004	0.8542 ± 0.0003
	w/o protos	0.8848 ± 0.0021	0.8845 ± 0.0018	0.8560 ± 0.0013	0.8561 ± 0.0009
	Local	0.8860 ± 0.0003	0.8850 ± 0.0005	0.8471 ± 0.0016	0.8461 ± 0.0012
	EnsKD	0.8846 ± 0.0014	0.8845 ± 0.0011	0.8553 ± 0.0006	0.8555 ± 0.0004
KMNIST	FedMCP++	0.8968 ± 0.0014	0.8966 ± 0.0014	0.8633 ± 0.0024	0.8633 ± 0.0024
	replicate	0.8938	0.8936	0.8614	0.8613
	w/o contrastive	0.8971 ± 0.0014	0.8969 ± 0.0014	0.8633 ± 0.0023	0.8633 ± 0.0023
	w/o protos	0.8972 ± 0.0017	0.8971 ± 0.0017	0.8642 ± 0.0024	0.8642 ± 0.0025
	Local	0.8925 ± 0.0007	0.8922 ± 0.0007	0.8397 ± 0.0017	0.8395 ± 0.0017
	EnsKD	0.8972 ± 0.0018	0.8970 ± 0.0018	0.8643 ± 0.0024	0.8642 ± 0.0024
STL-10	FedMCP++	0.3597 ± 0.0154	0.3089 ± 0.0148	0.2837 ± 0.0081	0.2249 ± 0.0099
	replicate	0.3670	0.3119	0.2845	0.2275
	w/o contrastive	0.3601 ± 0.0156	0.3102 ± 0.0158	0.2839 ± 0.0069	0.2257 ± 0.0084
	w/o protos	0.3653 ± 0.0126	0.3122 ± 0.0166	0.2835 ± 0.0065	0.2242 ± 0.0082
	Local	0.3639 ± 0.0079	0.3086 ± 0.0129	0.2645 ± 0.0036	0.1965 ± 0.0055
	EnsKD	0.3636 ± 0.0139	0.3126 ± 0.0160	0.2832 ± 0.0046	0.2239 ± 0.0067
CIFAR-10	FedMCP++	0.4874 ± 0.0069	0.4683 ± 0.0127	0.4318 ± 0.0047	0.4110 ± 0.0090
	replicate	0.4966	0.4795	0.4424	0.4226
	w/o contrastive	0.4877 ± 0.0069	0.4682 ± 0.0120	0.4328 ± 0.0034	0.4117 ± 0.0076
	w/o protos	0.4888 ± 0.0057	0.4696 ± 0.0110	0.4358 ± 0.0037	0.4153 ± 0.0079
	Local	0.5042 ± 0.0027	0.4879 ± 0.0036	0.4584 ± 0.0033	0.4415 ± 0.0032
	EnsKD	0.4894 ± 0.0040	0.4703 ± 0.0099	0.4352 ± 0.0029	0.4147 ± 0.0067

Note: method rows report five-seed mean ± standard deviation; replicate rows are same-seed re-runs of the original protocol (Section 5.2(e)).

**Table 9:** The obtained findings on six vision datasets with 30 clients. Metrics shown: global accuracy (GAcc), global F1 (GF1), client-local accuracy (CAcc), client F1 (CF1).

Dataset	Variant	GAcc	GF1	CAcc	CF1
MNIST	FedMCP++	0.9825 ± 0.0005	0.9824 ± 0.0005	0.9748 ± 0.0003	0.9746 ± 0.0004
	replicate	0.9828	0.9828	0.9748	0.9747
	w/o contrastive	0.9825 ± 0.0006	0.9824 ± 0.0006	0.9748 ± 0.0003	0.9746 ± 0.0003
	w/o protos	0.9825 ± 0.0004	0.9824 ± 0.0004	0.9750 ± 0.0004	0.9749 ± 0.0004
	Local	0.9826 ± 0.0004	0.9825 ± 0.0004	0.9717 ± 0.0004	0.9715 ± 0.0004
	EnsKD	0.9826 ± 0.0005	0.9826 ± 0.0005	0.9750 ± 0.0004	0.9749 ± 0.0004
SVHN	FedMCP++	0.4362 ± 0.0123	0.2688 ± 0.0146	0.3892 ± 0.0082	0.2367 ± 0.0090
	replicate	0.4335	0.2642	0.3826	0.2301
	w/o contrastive	0.4367 ± 0.0102	0.2700 ± 0.0144	0.3865 ± 0.0072	0.2348 ± 0.0084
	w/o protos	0.4399 ± 0.0112	0.2736 ± 0.0156	0.3936 ± 0.0085	0.2399 ± 0.0087
	Local	0.4526 ± 0.0116	0.2841 ± 0.0171	0.4047 ± 0.0073	0.2549 ± 0.0087
	EnsKD	0.4359 ± 0.0104	0.2683 ± 0.0152	0.3892 ± 0.0062	0.2357 ± 0.0075
Fashion-MNIST	FedMCP++	0.8739 ± 0.0014	0.8731 ± 0.0013	0.8428 ± 0.0011	0.8421 ± 0.0011
	replicate	0.8724	0.8721	0.8421	0.8418
	w/o contrastive	0.8737 ± 0.0022	0.8728 ± 0.0021	0.8425 ± 0.0011	0.8415 ± 0.0008
	w/o protos	0.8747 ± 0.0012	0.8739 ± 0.0012	0.8443 ± 0.0009	0.8436 ± 0.0013
	Local	0.8780 ± 0.0017	0.8763 ± 0.0016	0.8367 ± 0.0016	0.8347 ± 0.0016
	EnsKD	0.8740 ± 0.0014	0.8732 ± 0.0012	0.8442 ± 0.0011	0.8436 ± 0.0006
KMNIST	FedMCP++	0.8810 ± 0.0023	0.8809 ± 0.0023	0.8436 ± 0.0020	0.8437 ± 0.0021
	replicate	0.8805	0.8802	0.8423	0.8423
	w/o contrastive	0.8807 ± 0.0018	0.8805 ± 0.0018	0.8438 ± 0.0019	0.8439 ± 0.0019
	w/o protos	0.8818 ± 0.0021	0.8816 ± 0.0021	0.8447 ± 0.0023	0.8448 ± 0.0023
	Local	0.8762 ± 0.0019	0.8758 ± 0.0018	0.8145 ± 0.0019	0.8143 ± 0.0019
	EnsKD	0.8810 ± 0.0016	0.8809 ± 0.0016	0.8444 ± 0.0018	0.8444 ± 0.0018
STL-10	FedMCP++	0.3379 ± 0.0057	0.2823 ± 0.0085	0.2622 ± 0.0061	0.2038 ± 0.0081
	replicate	0.3212	0.2669	0.2508	0.1916
	w/o contrastive	0.3382 ± 0.0068	0.2833 ± 0.0093	0.2624 ± 0.0053	0.2041 ± 0.0073
	w/o protos	0.3392 ± 0.0085	0.2832 ± 0.0105	0.2630 ± 0.0031	0.2041 ± 0.0053
	Local	0.3412 ± 0.0024	0.2830 ± 0.0032	0.2356 ± 0.0023	0.1648 ± 0.0033
	EnsKD	0.3379 ± 0.0112	0.2824 ± 0.0122	0.2627 ± 0.0043	0.2036 ± 0.0074
CIFAR-10	FedMCP++	0.4391 ± 0.0068	0.4175 ± 0.0051	0.3634 ± 0.0087	0.3350 ± 0.0087
	replicate	0.4346	0.4070	0.3468	0.3141
	w/o contrastive	0.4388 ± 0.0084	0.4173 ± 0.0076	0.3638 ± 0.0091	0.3351 ± 0.0091
	w/o protos	0.4417 ± 0.0079	0.4204 ± 0.0064	0.3678 ± 0.0082	0.3411 ± 0.0083
	Local	0.4555 ± 0.0036	0.4336 ± 0.0062	0.3945 ± 0.0030	0.3709 ± 0.0049
	EnsKD	0.4399 ± 0.0071	0.4186 ± 0.0064	0.3654 ± 0.0089	0.3384 ± 0.0098

Note: method rows report five-seed mean ± standard deviation; replicate rows are same-seed re-runs of the original protocol (Section 5.2(e)).

**Table 10:** Parameter-averaging baselines under uniform partitioning (seed 0; 15 rounds; common broadcast initialization; Section 4.4). Worst = worst-client accuracy on the full test split; Gap = best-to-worst client gap. FedMCP++ uploads 41.6 KiB per client-round in every configuration; the baselines upload 411.0–413.5 KiB (grayscale) and 532.1–534.7 KiB (RGB). FedMCP++ rows report five-seed mean ± standard deviation (mean only for Worst and Gap; Section 5.2(e)); baseline rows are seed-0 runs.

Dataset	N	Method	GAcc	CAcc	Worst	Gap
MNIST	10	FedAvg	0.9899	0.9899	0.9899	0.0000
		FedPer	0.9837	0.9739	0.9675	0.0124
		FedRep	0.9885	0.9884	0.9880	0.0011
		FedMCP++	0.9884 ± 0.0004	0.9836 ± 0.0003	0.9822	0.0026
	20	FedAvg	0.9867	0.9867	0.9867	0.0000
		FedPer	0.9790	0.9692	0.9608	0.0151
		FedRep	0.9854	0.9853	0.9845	0.0013
		FedMCP++	0.9849 ± 0.0004	0.9786 ± 0.0003	0.9764	0.0047
	30	FedAvg	0.9852	0.9852	0.9852	0.0000
		FedPer	0.9733	0.9632	0.9446	0.0285
		FedRep	0.9836	0.9831	0.9824	0.0015
		FedMCP++	0.9825 ± 0.0005	0.9748 ± 0.0003	0.9716	0.0062
Fashion-MNIST	10	FedAvg	0.8972	0.8972	0.8972	0.0000
		FedPer	0.8808	0.8733	0.8658	0.0127
		FedRep	0.8886	0.8887	0.8875	0.0022
		FedMCP++	0.8956 ± 0.0023	0.8697 ± 0.0016	0.8636	0.0111
	20	FedAvg	0.8833	0.8833	0.8833	0.0000
		FedPer	0.8651	0.8573	0.8510	0.0142
		FedRep	0.8762	0.8754	0.8730	0.0040
		FedMCP++	0.8841 ± 0.0010	0.8543 ± 0.0006	0.8402	0.0238
	30	FedAvg	0.8726	0.8726	0.8726	0.0000
		FedPer	0.8481	0.8419	0.8187	0.0332
		FedRep	0.8615	0.8596	0.8559	0.0068
		FedMCP++	0.8739 ± 0.0014	0.8428 ± 0.0011	0.8243	0.0295
KMNIST	10	FedAvg	0.9284	0.9284	0.9284	0.0000
		FedPer	0.9141	0.9060	0.9018	0.0091
		FedRep	0.9231	0.9229	0.9215	0.0025
		FedMCP++	0.9183 ± 0.0004	0.8909 ± 0.0012	0.8862	0.0101
	20	FedAvg	0.9165	0.9165	0.9165	0.0000
		FedPer	0.8908	0.8807	0.8676	0.0233
		FedRep	0.9076	0.9072	0.9059	0.0029
		FedMCP++	0.8968 ± 0.0014	0.8633 ± 0.0024	0.8523	0.0189
	30	FedAvg	0.9008	0.9008	0.9008	0.0000
		FedPer	0.8829	0.8728	0.8641	0.0177
		FedRep	0.8940	0.8933	0.8911	0.0045
		FedMCP++	0.8810 ± 0.0023	0.8436 ± 0.0020	0.8311	0.0232
SVHN	10	FedAvg	0.7018	0.7018	0.7018	0.0000
		FedPer	0.6785	0.6775	0.6694	0.0122
		FedRep	0.7050	0.7038	0.6996	0.0084
		FedMCP++	0.6976 ± 0.0078	0.6486 ± 0.0065	0.6101	0.0664
	20	FedAvg	0.6260	0.6260	0.6260	0.0000
		FedPer	0.5302	0.5262	0.4967	0.0448
		FedRep	0.6075	0.6023	0.5921	0.0202
		FedMCP++	0.5570 ± 0.0083	0.5019 ± 0.0074	0.4484	0.1198
	30	FedAvg	0.4738	0.4738	0.4738	0.0000
		FedPer	0.3339	0.3349	0.3313	0.0197
		FedRep	0.4523	0.4451	0.4189	0.0427
		FedMCP++	0.4362 ± 0.0123	0.3892 ± 0.0082	0.3141	0.1449
CIFAR-10	10	FedAvg	0.6141	0.6141	0.6141	0.0000
		FedPer	0.5899	0.5863	0.5797	0.0125
		FedRep	0.5983	0.5947	0.5908	0.0098
		FedMCP++	0.5832 ± 0.0067	0.5289 ± 0.0075	0.4979	0.0544
	20	FedAvg	0.5274	0.5274	0.5274	0.0000
		FedPer	0.4880	0.4743	0.4607	0.0253
		FedRep	0.5075	0.5027	0.4978	0.0113
		FedMCP++	0.4874 ± 0.0069	0.4318 ± 0.0047	0.3620	0.1088
	30	FedAvg	0.4791	0.4791	0.4791	0.0000
		FedPer	0.4529	0.4285	0.4035	0.0392
		FedRep	0.4716	0.4588	0.4465	0.0225
		FedMCP++	0.4391 ± 0.0068	0.3634 ± 0.0087	0.2666	0.1484
STL-10	10	FedAvg	0.3675	0.3675	0.3675	0.0000
		FedPer	0.3282	0.2856	0.2569	0.0612
		FedRep	0.3451	0.2974	0.2732	0.0396
		FedMCP++	0.3894 ± 0.0063	0.3296 ± 0.0081	0.3030	0.0539
	20	FedAvg	0.3655	0.3655	0.3655	0.0000
		FedPer	0.2546	0.2237	0.1790	0.0934
		FedRep	0.2775	0.2334	0.1831	0.1030
		FedMCP++	0.3597 ± 0.0154	0.2837 ± 0.0081	0.2325	0.0955
	30	FedAvg	0.3519	0.3519	0.3519	0.0000
		FedPer	0.2436	0.1996	0.1547	0.0927
		FedRep	0.2450	0.2012	0.1689	0.0907
		FedMCP++	0.3379 ± 0.0057	0.2622 ± 0.0061	0.2005	0.1081

**Table 11:** The same comparison under Dirichlet (β = 0.3) label skew. CAcc (local) scores each client model on its own label distribution and CAcc (full) on the full test split (Section 3.4); Worst (local) is the worst client under the local protocol, reported because personalization can only manifest under the local protocol.

Dataset	N	Method	GAcc	CAcc (Local)	CAcc (Full)	Worst (Local)
MNIST	10	FedAvg	0.9857	0.9848	0.9857	0.9580
		FedPer	0.9762	0.9828	0.7309	0.9726
		FedRep	0.9781	0.9882	0.7668	0.9826
		FedMCP++	0.9736	0.9821	0.8142	0.9749
	20	FedAvg	0.9862	0.9862	0.9862	0.9792
		FedPer	0.9822	0.9756	0.6339	0.9314
		FedRep	0.9822	0.9816	0.6633	0.9332
		FedMCP++	0.9734	0.9716	0.7033	0.8872
	30	FedAvg	0.9815	0.9817	0.9815	0.9632
		FedPer	0.9740	0.9649	0.5529	0.9085
		FedRep	0.9784	0.9756	0.5903	0.9449
		FedMCP++	0.9698	0.9568	0.5942	0.7917
Fashion-MNIST	10	FedAvg	0.8793	0.8845	0.8793	0.8163
		FedPer	0.8519	0.9296	0.6348	0.8967
		FedRep	0.8479	0.9342	0.6314	0.8998
		FedMCP++	0.8586	0.9200	0.6726	0.8752
	20	FedAvg	0.8511	0.8434	0.8511	0.6527
		FedPer	0.8285	0.9125	0.5061	0.8276
		FedRep	0.8393	0.9213	0.5113	0.8318
		FedMCP++	0.8216	0.9039	0.5311	0.8008
	30	FedAvg	0.8611	0.8615	0.8611	0.7264
		FedPer	0.8375	0.8967	0.5139	0.7875
		FedRep	0.8462	0.9098	0.5362	0.7935
		FedMCP++	0.8200	0.8739	0.5345	0.7536
KMNIST	10	FedAvg	0.9141	0.9143	0.9141	0.8541
		FedPer	0.8930	0.9274	0.6389	0.9087
		FedRep	0.8961	0.9339	0.6614	0.9128
		FedMCP++	0.8809	0.8808	0.6403	0.8453
	20	FedAvg	0.8923	0.8931	0.8923	0.8679
		FedPer	0.8644	0.9130	0.5078	0.8692
		FedRep	0.8717	0.9242	0.5311	0.8908
		FedMCP++	0.8275	0.8443	0.4809	0.6697
	30	FedAvg	0.8822	0.8809	0.8822	0.8334
		FedPer	0.8681	0.8982	0.5141	0.8368
		FedRep	0.8701	0.9096	0.5445	0.8574
		FedMCP++	0.8058	0.7978	0.4598	0.4585
SVHN	10	FedAvg	0.6546	0.6072	0.6546	0.4435
		FedPer	0.6295	0.7265	0.3536	0.5815
		FedRep	0.6596	0.7785	0.3967	0.6613
		FedMCP++	0.4723	0.4819	0.2549	0.1361
	20	FedAvg	0.1596	0.1554	0.1596	0.0003
		FedPer	0.5658	0.7434	0.3304	0.5479
		FedRep	0.6068	0.7820	0.3652	0.6260
		FedMCP++	0.3808	0.5028	0.1844	0.0902
	30	FedAvg	0.1594	0.1458	0.1594	0.0000
		FedPer	0.4624	0.6477	0.2283	0.3557
		FedRep	0.5486	0.7179	0.2660	0.4950
		FedMCP++	0.2167	0.3546	0.1513	0.0062
CIFAR-10	10	FedAvg	0.5697	0.5706	0.5697	0.4598
		FedPer	0.5313	0.7274	0.3504	0.6430
		FedRep	0.5314	0.7279	0.3534	0.6465
		FedMCP++	0.4689	0.6117	0.3005	0.5265
	20	FedAvg	0.4970	0.4934	0.4970	0.3388
		FedPer	0.4636	0.6941	0.2917	0.6000
		FedRep	0.5019	0.7124	0.3120	0.6229
		FedMCP++	0.4550	0.6061	0.2640	0.4338
	30	FedAvg	0.5023	0.5029	0.5023	0.3126
		FedPer	0.4707	0.6996	0.2769	0.5314
		FedRep	0.4958	0.7135	0.2875	0.5454
		FedMCP++	0.4517	0.5856	0.2376	0.4221
STL-10	10	FedAvg	0.3494	0.3391	0.3494	0.1690
		FedPer	0.3150	0.5544	0.1814	0.4060
		FedRep	0.3059	0.5603	0.1883	0.4152
		FedMCP++	0.3306	0.5160	0.1723	0.3728
	20	FedAvg	0.3246	0.3080	0.3246	0.1294
		FedPer	0.1878	0.5684	0.1625	0.3903
		FedRep	0.2067	0.5749	0.1706	0.4068
		FedMCP++	0.2352	0.5285	0.1585	0.3388
	30	FedAvg	0.3155	0.3172	0.3155	0.0963
		FedPer	0.2225	0.4875	0.1484	0.3101
		FedRep	0.2430	0.4951	0.1526	0.3040
		FedMCP++	0.2540	0.4848	0.1564	0.3015

**Table 12:** Client-level fairness statistics under uniform partitioning: worst-client accuracy, best-to-worst gap, and across-client standard deviation of accuracy, reported as five-seed means from the released per-run files (Section 5.2(e)). Local = independent local training.

Dataset	N	Method	Worst	Gap	Client Std
MNIST	10	Local	0.9809	0.0031	0.0009
		FedMCP++	0.9822	0.0026	0.0008
	20	Local	0.9737	0.0055	0.0015
		FedMCP++	0.9764	0.0047	0.0012
	30	Local	0.9673	0.0084	0.0019
		FedMCP++	0.9716	0.0062	0.0015
Fashion-MNIST	10	Local	0.8538	0.0181	0.0056
		FedMCP++	0.8636	0.0111	0.0034
	20	Local	0.8288	0.0301	0.0081
		FedMCP++	0.8402	0.0238	0.0062
	30	Local	0.8166	0.0328	0.0080
		FedMCP++	0.8243	0.0295	0.0064
KMNIST	10	Local	0.8672	0.0172	0.0054
		FedMCP++	0.8862	0.0101	0.0031
	20	Local	0.8238	0.0293	0.0079
		FedMCP++	0.8523	0.0189	0.0046
	30	Local	0.7924	0.0390	0.0094
		FedMCP++	0.8311	0.0232	0.0055
SVHN	10	Local	0.6109	0.0656	0.0203
		FedMCP++	0.6101	0.0664	0.0204
	20	Local	0.4556	0.1219	0.0333
		FedMCP++	0.4484	0.1198	0.0303
	30	Local	0.3272	0.1542	0.0364
		FedMCP++	0.3141	0.1449	0.0335
CIFAR-10	10	Local	0.5076	0.0556	0.0156
		FedMCP++	0.4979	0.0544	0.0161
	20	Local	0.4308	0.0495	0.0123
		FedMCP++	0.3620	0.1088	0.0270
	30	Local	0.3442	0.0875	0.0214
		FedMCP++	0.2666	0.1484	0.0326
STL-10	10	Local	0.2880	0.0598	0.0175
		FedMCP++	0.3030	0.0539	0.0160
	20	Local	0.2259	0.0893	0.0231
		FedMCP++	0.2325	0.0955	0.0235
	30	Local	0.1915	0.0974	0.0263
		FedMCP++	0.2005	0.1081	0.0259

**Table 13:** Total runtime (in seconds) for 15 federated rounds for each method across six datasets using 10 clients.

Dataset	FedMCP++	Replicate	w/o Contrastive	w/o Protos	Local	EnsKD
MNIST	378.7	395.3	390.6	373.5	300.3	337.3
SVHN	1722.7	1713.0	1716.7	1681.3	1534.6	1608.2
Fashion-MNIST	379.2	377.5	377.5	370.0	313.0	345.1
KMNIST	396.4	396.0	396.2	379.7	314.4	345.6
STL-10	1213.7	1215.3	1214.1	1132.9	797.0	967.1
CIFAR-10	933.6	935.5	934.5	901.9	748.2	792.2

**Table 14:** Sensitivity to skew severity: β ∈ {0.1, 0.5} at 10 clients (seed 0; 15 rounds). CAcc (local) and CAcc (full) as defined in Section 3.4; the β = 0.3 values for FedMCP++ and FedAvg appear in Table 11.

Dataset	β	Method	GAcc	CAcc (Local)	CAcc (Full)
MNIST	0.1	FedAvg	0.9757	0.9767	0.9757
		Local	0.8698	0.9828	0.4760
		FedMCP++	0.9127	0.9364	0.4770
	0.5	FedAvg	0.9885	0.9886	0.9885
		Local	0.9857	0.9825	0.8183
		FedMCP++	0.9846	0.9836	0.9016
Fashion-MNIST	0.1	FedAvg	0.8463	0.8504	0.8463
		Local	0.6786	0.9663	0.3734
		FedMCP++	0.6958	0.9409	0.3631
	0.5	FedAvg	0.8896	0.8882	0.8896
		Local	0.8868	0.9085	0.7142
		FedMCP++	0.8796	0.8903	0.7896
KMNIST	0.1	FedAvg	0.8700	0.8706	0.8700
		Local	0.7555	0.9548	0.3524
		FedMCP++	0.7580	0.9118	0.3306
	0.5	FedAvg	0.9156	0.9164	0.9156
		Local	0.9107	0.9025	0.7046
		FedMCP++	0.8976	0.8809	0.7677
SVHN	0.1	FedAvg	0.1594	0.1021	0.1594
		Local	0.4940	0.8276	0.2219
		FedMCP++	0.2156	0.5485	0.1152
	0.5	FedAvg	0.6953	0.6730	0.6953
		Local	0.6776	0.6790	0.3622
		FedMCP++	0.5563	0.4885	0.3162
CIFAR-10	0.1	FedAvg	0.5464	0.5786	0.5464
		Local	0.4921	0.7810	0.2746
		FedMCP++	0.3555	0.6547	0.2387
	0.5	FedAvg	0.5540	0.5434	0.5540
		Local	0.5799	0.6841	0.3318
		FedMCP++	0.4985	0.6125	0.3151
STL-10	0.1	FedAvg	0.2799	0.2457	0.2799
		Local	0.2853	0.6965	0.1641
		FedMCP++	0.2263	0.6748	0.1560
	0.5	FedAvg	0.3759	0.3613	0.3759
		Local	0.3470	0.4723	0.2035
		FedMCP++	0.3230	0.4569	0.2036

**Table 15:** One hundred clients under uniform partitioning (seed 0; 15 rounds; per-client samples: 600 on the grayscale benchmarks, 732 on SVHN, 500 on CIFAR-10; STL-10 excluded by design, Section 5.2(b)). The per-client uplink of FedMCP++ is unchanged at 41.6 KiB per round.

Dataset	Method	GAcc	CAcc	Worst	Gap
MNIST	Local	0.9704	0.9487	0.9358	0.0222
	FedMCP++	0.9674	0.9534	0.9435	0.0170
Fashion-MNIST	Local	0.8297	0.7902	0.7421	0.0684
	FedMCP++	0.8135	0.7852	0.7282	0.0817
KMNIST	Local	0.8076	0.7135	0.6777	0.0663
	FedMCP++	0.7942	0.7350	0.6925	0.0613
SVHN	Local	0.1959	0.1994	0.1469	0.1204
	FedMCP++	0.1959	0.2003	0.1585	0.1050
CIFAR-10	Local	0.4214	0.3265	0.2510	0.1420
	FedMCP++	0.4134	0.3411	0.2676	0.1167

**Table 16:** Proxy sensitivity of FedMCP++ (10 clients, seed 0, 15 rounds). Rows vary the public-proxy size; “disjoint” excludes the proxy samples from the evaluation split, so no evaluated sample is ever seen during distillation. The 1000-sample overlap rows are the corresponding cells of Table 10.

Dataset	Proxy Size	GAcc	CAcc
Fashion-MNIST	250	0.8991	0.8663
	500	0.8992	0.8702
	1000	0.8986	0.8710
	2000	0.8967	0.8748
	1000 (disjoint)	0.8989	0.8703
CIFAR-10	250	0.5868	0.5258
	500	0.5882	0.5237
	1000	0.5780	0.5281
	2000	0.5793	0.5354
	1000 (disjoint)	0.5792	0.5295

**Table 17:** Membership-inference evaluation: mean Yeom confidence-attack AUC over clients (0.5 = no leakage; 10 clients, seed 0, up to 500 member and 500 non-member samples per client).

Dataset	FedAvg	Local	FedMCP++
MNIST	0.4943	0.5270	0.5235
Fashion-MNIST	0.5113	0.5503	0.5510
KMNIST	0.6277	0.7154	0.7041
SVHN	0.4311	0.4379	0.4317
CIFAR-10	0.4610	0.4744	0.4661
STL-10	0.4665	0.5064	0.4882

## Data Availability

The data supporting the findings of this study are openly available public benchmark datasets: MNIST, Fashion-MNIST, KMNIST, SVHN, CIFAR-10, and STL-10. No new data were created in this study. The complete implementation used for this study and its revision experiments (fedmcp_v2), including the Local, EnsKD, FedAvg, FedPer, and FedRep baselines, Dirichlet partitioning, fairness logging, proxy-sensitivity options, multi-seed aggregation, and the membership-inference evaluation, is publicly released, together with the completed verification cells reported in Section 4.4 and Section 5.2 (Fashion-MNIST, 10 clients: the baseline comparison under both partitionings, the five-seed replication, and the membership-inference measurement, together with the complete four-baseline MNIST-10 cell), at https://github.com/fbozkurt/FedMCP-plus-plus-v2 (accessed on 14 August 2026).

## Abbreviations

The following abbreviations are used in this manuscript:

FL	Federated Learning
non-IID	Non-Independent and Identically Distributed
IoT	Internet of Things
CNN	Convolutional Neural Network
KD	Knowledge Distillation
KL	Kullback–Leibler (divergence)
MSE	Mean Squared Error
NT-Xent	Normalized Temperature-scaled Cross-Entropy (loss)
SGD	Stochastic Gradient Descent
GAcc/GF1	Global Accuracy/Global F1-score
CAcc/CF1	Client-local Accuracy/Client-local F1-score
FLOPs	Floating-Point Operations
PFL	Personalized Federated Learning
EnsKD	Ensemble Knowledge Distillation (baseline)
MIA	Membership-Inference Attack
